# Global insight into rare disease and orphan drug definitions: a systematic literature review

**DOI:** 10.1136/bmjopen-2024-086527

**Published:** 2025-01-25

**Authors:** Ghada Mohammed Abozaid, Katie Kerr, Hiba Alomary, Hussain A Al-Omar, Amy McKnight

**Affiliations:** 1 Centre for Public Health, Institute of Clinical Sciences B, Royal Victoria Hospital, Queen's University Belfast School of Medicine, Dentistry and Biomedical Sciences, Belfast, UK; 2 Pharmacy Practice, Princess Nourah bint Abdulrahman University, Riyadh, Saudi Arabia; 3 Department of Applied Linguistics, Princess Nourah bint Abdulrahman University, Riyadh, Saudi Arabia; 4 Department of Clinical Pharmacy, College of Pharmacy, King Saud University, Riyadh, Saudi Arabia

**Keywords:** EPIDEMIOLOGIC STUDIES, Health policy, Public health, Review, Systematic Review, GENETICS

## Abstract

**Abstract:**

**Objectives:**

This study sheds light on the available global definitions, classifications, and criteria used for rare diseases (RDs), ultrarare diseases (URDs), orphan drugs (ODs) and ultraorphan drugs (UODs) and provides insights into the rationale behind these definitions.

**Design:**

A systematic literature review was conducted to identify existing definitions and the criteria used to define RDs, ODs and their subtypes.

**Data sources:**

Searches were performed in the PubMed/Medline, Embase, Scopus and Web of Science (Science and Social Sciences Citation Index) databases covering articles published from 1985 to 2021.

**Eligibility criteria for selecting studies:**

English-language studies on the general human population were included if they provided definitions or criteria for RDs, ODs and/or their subtypes without restrictions on publication year, country or jurisdiction.

**Data extraction and synthesis:**

Two independent reviewers conducted the search, screening and data extraction. Narrative synthesis, content analysis and descriptive analyses were conducted to extract and categorise definitions and criteria from these sources. Study quality was assessed using the Joanna Briggs Institute (JBI) critical appraisal tools.

**Results:**

Online searches identified 2712 published articles. Only 93 articles met the inclusion criteria, with 209 distinct definitions extracted. Specifically, 93 of these articles pertained to 119 RDs, 11 URDs, 67 ODs and 12 UODs. These definitions varied in their reliance on prevalence based and other contextual criteria.

**Conclusion:**

Prevalence-based criteria alone pose challenges, as disease frequencies differ by country. Establishing country-specific definitions can enhance understanding, support intercountry evaluations, improve healthcare efficiency and access to ODs, and strengthen equity and equality in healthcare. Such efforts would also promote research and development and support better outcomes for patients with complex and rare conditions.

**PROSPERO registration number:**

CRD42021252701.

**STRENGTHS AND LIMITATIONS OF THIS STUDY**

- This systematic literature review, based on PROSPERO International Prospective Register of Systematic Reviews (CRD42021252701) and Preferred Reporting Items for Systematic Review and Meta-Analysis Protocols (PRISMA-P), explores criteria for determining rare diseases (RDs) and orphan drugs (ODs) without publication design, year or regional restrictions.
- Unlike other reviews, this study explored different criteria for defining RDs and ODs issued by different agencies and entities to fulfil their mandates in relation to RDs and ODs.
- The searched articles showed inconsistent terminology, and despite seeking library specialist feedback, some relevant studies might have been missed.
- The results might be subject to biases in publication selection, language and database.
- A limitation of this study is that it relies only on literature-based definitions, which may not fully capture the regulatory definitions officially adopted by agencies, despite these being the ones directly applicable in real-world situations.

## Background

 Rare diseases (RDs) represent a major public health concern requiring more effective interventions to alleviate the burden on patients, carers, health and social care systems. RDs, sometimes known as ‘orphan diseases’^1 2^ and affect a minority of people, are typically medical conditions that are individually identified with low prevalence within a particular population.^3^ Globally, RDs affect more than 450 million individuals,^4^ the majority of whom are disproportionately disadvantaged and lack effective treatment. No multipurpose and universally agreed on definition of a RD^5^ exists, making optimal care difficult; definitions implemented internationally each depend on the context and perspectives of various stakeholders, some of which employ qualitative and/or quantitative criteria.^6^

The qualitative criteria used to define RDs are primarily subjective and include terms such as ‘life-threatening’, ‘alternative treatment options’, ‘severity of disease’, and ‘neglected’. Some of these criteria have major emotional impacts, such as the severity of the illness, its potential fatality, heritability, or the lack of effective therapies.^7^ On the other hand, quantitative criteria to define RDs are objective and measurable in nature and include disease incidence^8^ and prevalence,^9^ which are key indicators for understanding the frequency of disease occurrence within a population. Certain diseases can be labelled rare in one nation but not in another owing to population genetic variations, environmental or societal influences, or disparities in survival rates across different regions.^10^ A lack of sufficient data on which diseases are categorised as rare creates an obstacle in understanding these conditions and proportions and disease coding; ensuring accurate diagnoses; and encouraging pharmaceutical companies^11^ to invest in the research and development of medications for these diseases and manufacture orphan drugs (ODs), which, consequently, constitute a considerable challenge in making treatments available and accessible.

Overall, effective therapies are available for fewer than 5% of individuals diagnosed with RDs. The definition of RD is used to determine the eligibility of a medication for a regulatory designation as an OD. This is a status granted to pharmaceutical products that are developed to treat RDs and incentivised by governments and regulatory bodies to encourage product development and production. For instance, pricing preferences, market exclusivity, financial incentives, protocol assistance, grants and research funding, and extended patent protection are different forms of incentives offered to industry.

OD definitions extend across international borders and are frequently linked to RD definitions that are based on epidemiological data for the target disease and economic data for the drug market.^5^ Some countries set priorities for RD expenditures and resource allocation to address OD accessibility and help policy-makers enhance the efficiency and delivery of ODs.^6^ Adopting a universal definition can be challenging due to regional variations in terms of demographic, economic, survival and sociocultural factors.^7^ For example, in Saudi Arabia (SA), there is no multipurpose national definition for RD or OD, which could impact diagnoses, treatment strategies, and resource allocation, highlighting the need for a localised and country-specific definition. Approximately 80% of RDs have a genetic cause, which increases the risk of inherited autosomal conditions in offspring from consanguineous marriages;^12^ in SA, 70% of total marriages are consanguineous, which may increase the prevalence of some genetic diseases.^13^

There are considerable challenges associated with the context and practical use of RDs, ODs and subtype definitions employed by various stakeholders. One significant challenge is the inconsistency in definitions across regions and regulatory agencies. For example, the European Union (EU) and the US use different prevalence thresholds to define RDs, complicating regulatory frameworks and market access for ODs. This variation also affects clinical trials and research, as the lack of harmonised definitions can hinder data comparability and international collaboration. Moreover, pharmaceutical companies face additional regulatory and pricing barriers due to these differences, which can delay drug approval and patient access. From a patient care perspective, disparities in definitions may lead to inequities in diagnosis, treatment and access to therapies. ODs may not be available to patients in other regions with the same condition, fragmenting advocacy efforts. Finally, economic and ethical considerations, such as cost-effectiveness criteria and the financial burden on healthcare systems, further complicate the practical use of the RD and OD definitions, highlighting the need for harmonisation to ensure equitable and efficient healthcare delivery globally for patients with RD.

This systematic literature review (SLR) explores the diverse definitions and criteria used by countries to define RDs, ODs and their subtypes, providing deeper insight into different factors, encouraging the establishment of robust criteria, and supporting policy deliberations.

## Methods

### SLR protocol

The protocol for this SLR^11^ was registered with the PROSPERO International Prospective Register of Systematic Reviews (CRD42021252701) and follows the Preferred Reporting Items for Systematic Review and Meta-Analysis Protocols (PRISMA-P)^14 15^ guidelines. The PROSPERO template ensures transparency and accountability for SLRs, while the PRISMA-P provides a flow chart for the identification, screening, eligibility and inclusion phases of the review process.

### Search strategy

The PubMed/Medline, Embase, Scopus and Web of Science (Science and Social Sciences Citation Index) databases were queried to answer the research question ‘What are the criteria for defining RDs, ultrarare diseases (URDs), ODs, and ultraorphan drugs (UODs) globally?’ as in online supplemental table 1. The search strategies and terms used were identified based on specific inclusion and exclusion criteria. The inclusion criteria included patients with RD receiving ODs. The publication year, country and jurisdiction were not restricted. Studies that were published in English and provided data for the general human population were included.

The exclusion criteria included rare cancers, infectious diseases, poisonings,^11^ studies focused on specific RDs or ODs, non-English language studies and non-human studies. The decision to restrict the search to English-language studies were based on several considerations. First, the majority of high-impact journals publish in English, which is the primary language for scientific communication worldwide. Limiting the search to English ensures that we capture the most relevant and widely recognised studies. Second, the scarcity of resources for translating non-English articles, coupled with the potential for errors when using automatic translation tools, could potentially compromise the reliability and accuracy of data extraction and synthesis processes. Furthermore, language constraints in systematic reviews generally have little effect on the overall conclusions, especially in fields where English-language publications dominate the literature. For RDs and ODs in particular, the concentration of research and policy discussions in English-speaking or international journals is significant. Restricting the search to English enables a practical, targeted evaluation while maintaining scientific rigour.

Rare cancers were excluded from this review to maintain a focused scope and ensure that the analysis remained manageable and relevant to the broader definitions of RDs and ODs. Rare cancers often follow distinct clinical, regulatory and research frameworks compared with non-cancerous RDs. These include oncology-specific diagnostic criteria, treatment pathways and regulatory incentives such as OD designation. Including rare cancers would have introduced complexity, potentially detracting from the broader analysis of non-cancerous RDs and ODs. Additionally, rare cancers are frequently treated as a separate category in both regulatory contexts and the literature. Their exclusion aligns with the rationale detailed in the published protocol.^11^

The identified articles subsequently underwent both forward and reverse citation screening. The initial search was conducted in 2021. To ensure the review included the most recent and pertinent studies, updated searches were performed on 31 December 2022 and 31 December 2023. These updates represent a methodological refinement to the original protocol and were undertaken to capture contemporary studies published after the initial search period. This approach reflects our commitment to ensuring comprehensive coverage of relevant literature and providing the most up-to-date evidence in the analysis.

### Patient and public involvement

Patients or members of the public were not involved in the design, conduct, reporting, or dissemination plans of this research.

### Study selection and data extraction

After searching the different databases, studies were selected, and duplicates were removed. To determine the initial eligibility of the studies based on the inclusion and exclusion criteria,^11^ two rounds of abstract and title screening were performed by two reviewers (GMA and KK) independently. A third reviewer (AM) arbitrated any disputes between GMA and KK, and all decisions were recorded in a Microsoft Excel spreadsheet. Likewise, for full-text screening, if there were instances of missing or unreported data or if further details were necessary, GMA reached out to the study author(s) to request missing data. The timeframe for a response before excluding the article due to insufficient information was set at 3 weeks.

The extracted data encompassed various elements, including author names, publication information, journal title, study design, organisation, country, quality assessment and reference definitions of RDs and ODs. Additionally, these data encompassed qualitative and/or quantitative criteria used to define RDs, ODs and their subtypes. The qualitative criteria considered disease features, intended drug use, patient group, therapeutic impact and regulatory support, offering a comprehensive view beyond numerical values. The quantitative criteria considered numerical thresholds pivotal for regulation, science and policies, providing precise metrics based on disease prevalence and target demographics. Moreover, the extracted data involved the underlying reasoning for each definition, the status of the definition, and whether the RD and OD definitions were considered by reviewers independently using the Covidence platform, a web-based platform for conducting SLRs.^16 17^

### Quality assessment

The study quality was assessed by GA and KK using the Joanna Briggs Institute (JBI) critical appraisal tools^18^ to evaluate the trustworthiness, relevance and outcomes of published studies conducted independently using a Microsoft Excel spreadsheet.

### Data analysis

A narrative synthesis summarising the data from the included studies was performed. The preliminary synthesis involved content analysis of the qualitative data, with coding employed to explore themes. Descriptive statistics were performed and included frequencies and percentages to report and summarise the quantitative criteria from the included studies. This process was intended to illustrate the key themes and numerical information presented in these definitions by using two independent coders (GMA and HiA) with different backgrounds; conflicts were resolved through collaborative discussion. The analyses aimed to identify key elements defining RDs, URDs, ODs and UODs qualitatively and quantitatively.

## Findings

### PRISMA and quality assessment

**Figure 1:**
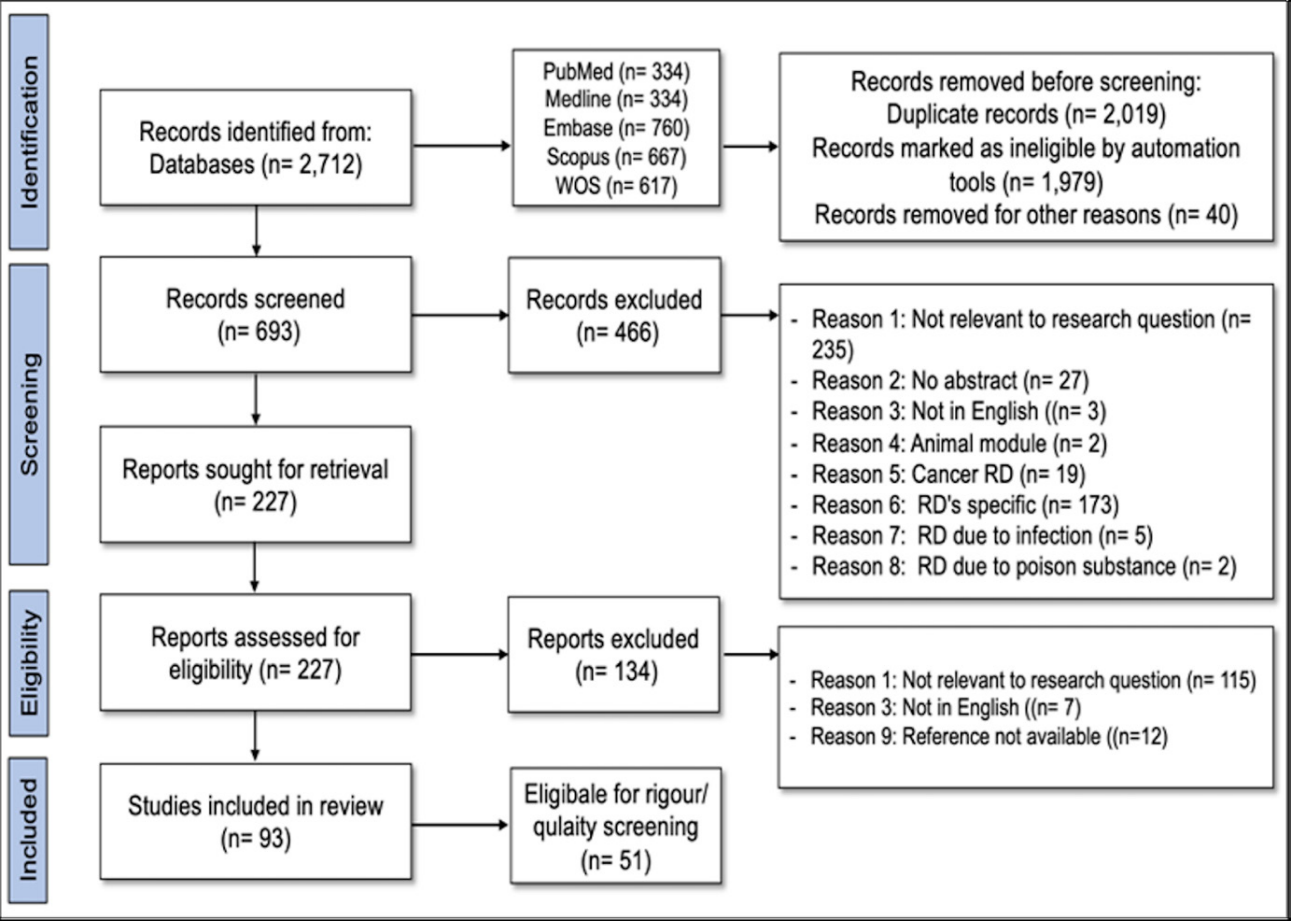
Description of Preferred Reporting Items for Systematic Reviews and Meta-Analyses flow chart. RDs, rare diseases; WOS, Web of Science.

The initial search yielded 2712 studies identified from different databases. The published articles spanned from 1985 to 2021. A total of 2019 articles were duplicates and were removed; for example, title and abstract screening excluded 466 studies, and 235 studies were recorded as not relevant to the SLR research questions due to a lack of abstracts (n=27) or were not in English (n=3); instead, they focused on non-human (n=2), cancer-related RDs (n=19), specific RDs (n=173), or infections (n=5) or poisonings (n=227) (online supplemental table 2). The final review included 93 studies whose full texts were retrieved (figure 1).

A total of 93 articles met the inclusion criteria, and 209 distinct definitions were extracted. Specifically, 93 of these articles mentioned RDs, 11 URDs, 67 ODs and 12 UODs. Fifty-one studies were considered in the final quality assessment. A full list of included studies is provided in online supplemental table 3. Likewise, the critical appraisal results for systematic reviews and research syntheses, economic evaluations, text opinion studies, analytical cross-sectional studies, qualitative research, prevalence studies and cohort studies were outlined and provided in online supplemental table 4.

### Geographical overview of the definitions

**Figure 2:**
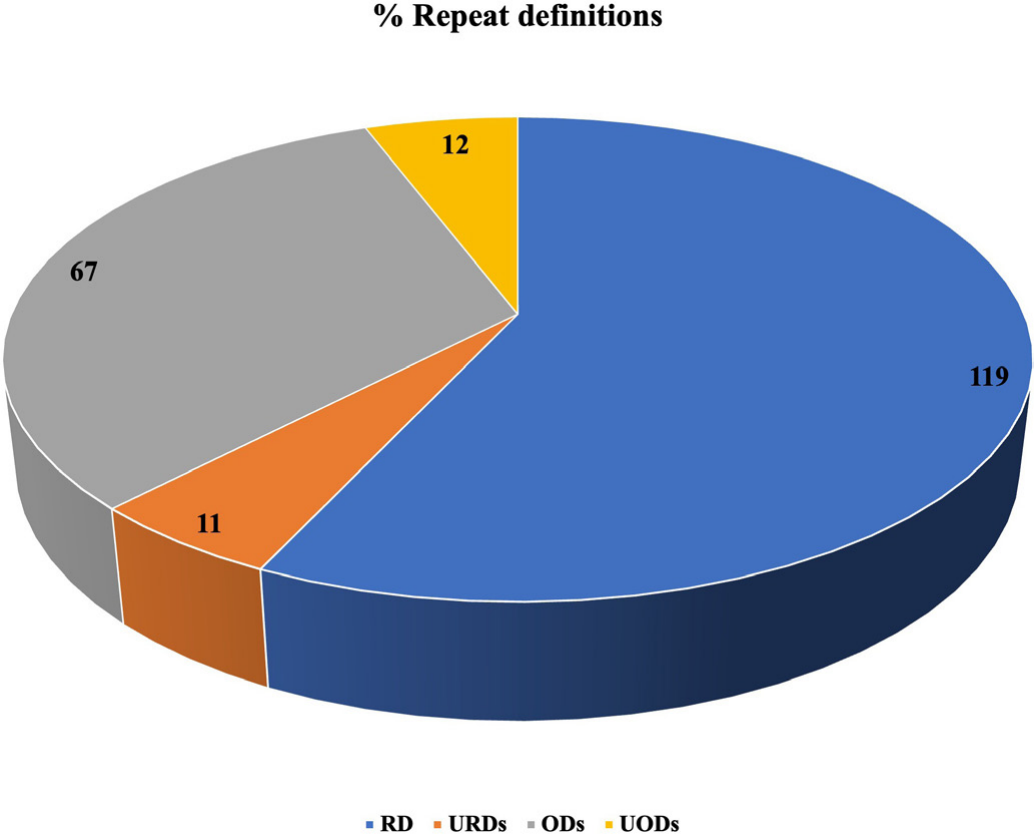
Description of repeated definitions included in the studies. ODs, orphan drugs; RD, rare disease; UODs, ultraorphan drugs; URDs, ultrarare diseases.

A total of 209 definitions were identified in the 93 included articles; these were for RDs (n=119, 56.93%); URDS (n=11, 5.26%); ODs (n=67, 32.06%); and UODs (n=12, 5.75%) (figure 2).

RD and OD definitions were often linked. Nonetheless, the most frequent definition employed for RDs, and ODs was the EU definition, accounting for approximately 40% and 24%, respectively, of the cases. EU nations employ both qualitative and quantitative criteria to define RDs as ‘diseases that are life-threatening or chronically debilitating illnesses with extremely low prevalence (less than 5 per 10,000)’.^19 20^ Similarly, the US Food and Drug Administration (FDA) defines RDs as ‘any ailment or condition that impacts fewer than 200 000 individuals in the USA or that affects over 200 000 people in the USA, with no foreseeable likelihood of recuperating the expenses associated with developing and providing a drug for such a disease or condition through sales of the drug in the USA’.^21 22^ An OD in the EU is typically defined as ‘a pharmaceutical product for diagnosing, preventing, or treating a rare disease’.^23^

The geographical analysis presented in this SLR examined the global distribution of RD (online supplemental table 5), OD (online supplemental table 6), URD (online supplemental table 7) and UOD (online supplemental table 8) criteria used to define them across different geographical regions.

### RD definitions

In Europe, 48 studies discussed RD definitions. Specifically, the EU (36), the UK (3), Germany (1), Latvia (1), the Netherlands (1), Poland (2), Romania (1), France (2) and Ukraine (1) had studies that defined RDs as diseases with a prevalence of 5 or fewer cases per 10 000 individuals. The UK defines RDs based on a prevalence threshold of fewer than 1 in 2000 people. In Eastern Europe and Northern Asia, Russia had one article; in Southeast Europe, Southwestern Europe and Asia, Turkey had an article discussing RD definitions, both showcasing differences in prevalence thresholds compared with the EU definition.

In North America, 28 studies were identified, 24 from the USA and two from Canada. The USA defines RDs based on a prevalence of less than 200 000 individuals living with a RD. In addition, the Rare Disease Act defines RDs based on qualitative criteria indicating that it occurs so infrequently in the USA that there is no reasonable expectation for the cost of developing and making a drug available in the USA for such a disease or condition to be recuperated from its sales. However, the Canadian Organization for Rare Disorders suggested that 1 in 12 Canadians, approximately 2.8 million individuals, might be living with an RD. South America contributed two studies—one from Chile and one from Peru—where RDs were defined by disease severity, categorising them as ‘life-threatening’ and ‘severely or chronically debilitating’ (online supplemental table 5).

Oceania had differing prevalence thresholds according to RD definitions: Australia (10) and New Zealand (1) used a disease prevalence of 1.1 per 10 000 individuals. Australia has established a prevalence rate of 1.16 per 100 000 individuals for an RD. The prevalence threshold for orphan disease designation is 0.9 in 10 000 individuals. The estimated incidence rate is 1 in 10 000 individuals in Australia.

Asian countries (Japan, Taiwan, China, South Korea, Singapore, India, Armenia and the Philippines) each defined RDs based on varying criteria such as prevalence rates, genetic disorders, disease severity and incidence thresholds (online supplemental table 5).

In Africa, Egypt and Kenya were the only countries to mention and discuss RD definitions based on specific conditions and disease severity.

**Figure 3:**
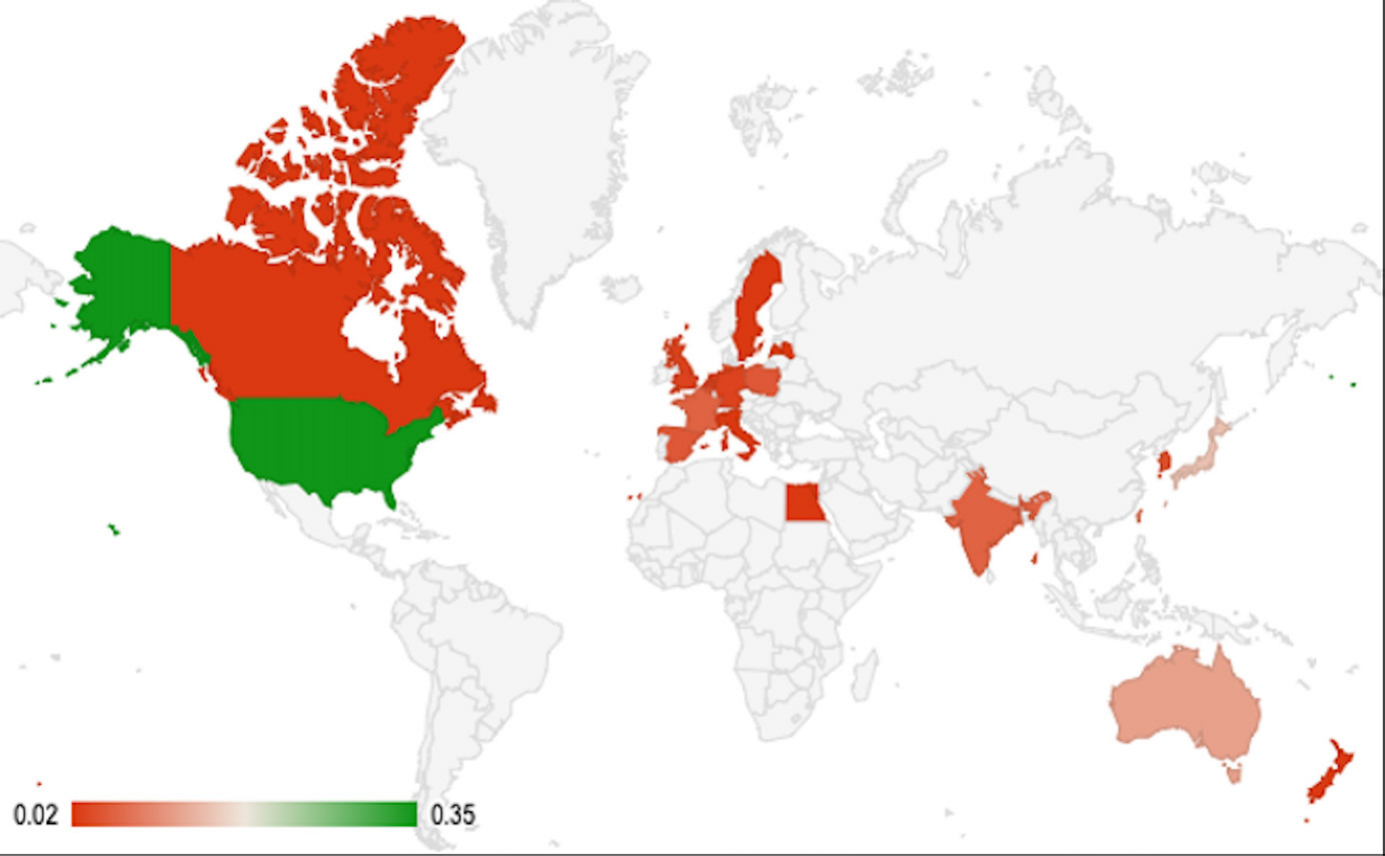
Global insight into rare disease prevalence (dark red indicates low prevalence, and dark green indicates greater prevalence).

The majority of the definitions extracted were from Europe [EU (43%), the UK (22%), France (6%), Poland (5%), Spain (5%), Belgium (4%), Germany (3%), the Netherlands (3%), England (3%), Scotland (3%), Lativa (2%), Italy (2%) and Sweden (2%)], followed by North America [US (35%) and Canada (2%)] and Asia and Oceania [Japan (15%), Australia (12%), Taiwan (9%), India (6%), South Korea (4%), New Zealand (2%) and Singapore (2%)]. Global perspectives on RD definitions from the WHO and Orphanet revealed further variations in prevalence thresholds and disease severity criteria (figure 3). A summary of RDs definitions is provided based on the country provided in table 1.

**Table 1:** A summary of RDs definitions is provided based on the country

Country, frequency	# of articles; (%)		(RD) definition	Date
USA (25)	24(26%)	Orphan Drug Regulation	Defines RD according to prevalence: ‘‘rare disease’ means any disease or condition that affects less than 200 000 persons in the USA’.	1993
		RDA		2002
		ODA	Defined RDs based on qualitative descriptors as follows: ‘the term ‘rare disease or condition’ means any disease or condition which occurs so infrequently in the USA that there is no reasonable expectation that the cost of developing and making available in the USA a drug for such disease or condition will be recovered from sales in the USA of such drug’.	1983
		FDA	Define RD as ‘any disease or condition that affects less than 200 000 people in the USA or affects >200 000 in the USA and for which there is no reasonable expectation that the cost of developing and making available in the USA a drug for such disease or condition will be recovered from sales in the USA of such drug’	
Canada (3)	2(2%)	CORD	Rare disease as one that afflicts less than 1 person in 200 000.	
			Estimated that 1 in 12 Canadians, or about 2.8 million individuals, may be living with a rare disease	
UK (3)	2(2%)	the Rare Disease Framework	Defined RD based on prevalence, as a condition affecting fewer than 1 in 2000 people. (ie, a prevalence of 5 or less per 10,000)	2021
		NHS	Some countries use additional definitions in situations where a condition is not officially defined as rare. classifies all conditions that require specialized medical care as rare if they occur in <500 citizens yearly	
EU (36)	35(38%)		Rare diseases, including those of genetic origin, are life-threatening or chronically debilitating diseases which are of such low prevalence (less than 5 per 10 000 persons in the European Union) that special combined efforts are needed to address them so as to prevent significant morbidity or perinatal or early mortality or a considerable reduction in an individual’s quality of life or socio-economic potential.	
		European Commission on Public Health	Defines rare diseases as ‚life-threatening or chronically debilitating diseases which are of such low prevalence that special combined efforts are needed to address them.	
		Orphan Drug Regulation	A disease or disorder that affects fewer than 5 in 10 000 citizens is the definition for rare	141/2000
		EMA	prevalence of rare disease <5/10 000	
France (2)	2(2%)		Affect fewer than 1 in 2000 (ie, a prevalence of 5 or less per 10,000)	
Japan (13)	13(14%)		Japan diseases with a prevalence of 4.0/10,000	
			<50 000 patients in Japan	
			Intractable diseases‚ is a Japan-specific conception of diseases with (i) unknown etiology (ii) no effective treatment, (iii) rare status (iv) necessity of long-term treatment	
			The incidence rate is estimated to be ≤2.5 cases in 10 000 for Japan	
Taiwan (7)	7(8%)	Taiwan Foundation for Rare Disorders	Diseases affecting <1 in 10 000 that are officially recognized are eligible for medical coverage.	2000
		Physically and Mentally Disabled Citizens Protection Act	RD is one type of disability	2001
China (5)	5(5%)	the Chinese Society of Genetic Medicine	Genetic disorders affect with less than one over 50 000 of the incidences in Newborn babies.	
			Incidence of the disease in adults or neonates is less than 1 in 500 000 and 1 in 10,000, respectively.	
South Korea (4)	5(5%)		Prevalence thresholds have been set at less than 1 per 20 000	
			Prevalence threshold: <4.0 in 10 000	
			< 20 000 people in Korea (ie, <4 per 10 000 population)	
WHO (5)	5(5%)		Rare disease affects at most 6.5 out of every 10 000 individuals.	
			Frequency of 6.5–10/10 000 inhabitants	
			Incidence ranges approximately from 0.65–1% in the whole population.	
			Rare disease as affecting 65/100 000–100/100 000 persons.	
Orphanet (1)	1(1%)		Disease inventory, it is evident that the majority of RDs are of genetic etiology, and a smaller percentage is autoimmune or infectious disorders, in addition to some rare cancers.	

CORDThe Canadian Organization of Rare DiseasesEMAEuropean Medicines AgencyEUEuropean UnionFDAThe Food and Drug AdministrationNHSNational Health ServiceODAThe Orphan Drug ActRDAThe Rare Diseases Act

### URD definitions

The definitions of URDs primarily originated from the European continent, encompassing the UK, Poland and North America, and including, for example, Alberta and Ontario; URDs typically affect ≤1 in 50 000 or fewer individuals within a population. Additional criteria for classifying URDs varied by region and authority. The Advisory Group for National Specialized Services stipulates that in England, the prevalence should be less than 500 individuals affected (~2500/100 000 of the population). The National Institute for Health and Care Excellence (NICE) further narrows this definition, classifying URDs as those with a prevalence of ≤1:50 000 people. Ontario employs a criterion of fewer than 1 in 150 000 live births or new diagnoses per year, while the definition in Poland aligns with the EU definition, designating URDs as affecting fewer than 1 in 50 000 people. URDs may also be termed ‘singular cases’ or ‘individual cases,’ given their exceptionally low prevalence (online supplemental table 7). Based on the country, a summary of URDs definitions is provided in table 2.

**Table 2:** A summary of ultrarare diseases (URDs) definitions is provided based on the country

Country, frequency		(URD) definition
UK		Ultra-orphan diseases, the term refers to chronic diseases with a prevalence of 1 in 50 000 of the population (Hughes *et al*,^39^ 2005)
	NICE	Ultra-orphan diseases affect a very small patient population, defined by the National Institute for Health and Care Excellence (NICE) as those diseases with a prevalence of ≤1: 50 000
England	Advisory Group on National Specialized Services (AGNSS)	The qualifier required by AGNSS was less than 500 persons affected in England (ie, ∼1 in 100 000 of the English population)
Ontario		An incidence rate of fewer than 1 in 150 000 live births or new diagnoses per year in Ontario
England and Wales	NICE	Ultra-orphan conditions are defined as diseases affecting <1000 people in England and Wales by the National Institute for Health and Care Excellence (NICE)

### OD definitions

Nineteen studies described OD definitions within Europe, with one from Italy and another from Germany both adopting the European Medicines Agency (EMA) definition, indicating that a drug can be defined as an OD if it is intended for the diagnosis, prevention or treatment of life-threatening or chronically serious debilitating conditions affecting no more than 5 in 10 000 individuals. Similarly, one study from Italy followed the Italian Medicines Agency criteria, focusing on three aspects: unmet medical needs, clinical added value and quality of evidence. Moreover, one study from Germany suggested that specific health technology assessment criteria be used for the definition of ODs; these criteria are associated with higher p values when sample sizes are limited, when surrogate endpoints are used, when therapeutic benefit is added, and when the annual budget impact for a given indication is less than €50 million.

In North America, there were nine studies, all of which aligned with the USA FDA regulations, indicating that an OD represents a condition affecting fewer than 200 000 persons in the USA or meets the cost recovery provisions.

In Asia, six studies described ODs, one from Singapore, one from Vietnam and two from China, all of which contributed to the body of evidence on ODs. It was also reported in two studies that the OD Centre in Korea provides medications for diseases affecting fewer than 1 in 20 000 individuals. These encompass illnesses lacking adequate treatments or drugs or drugs that notably enhance safety or efficacy compared with existing alternatives. In contrast, in China, ODs are characterised by their availability as pharmaceutical products or active ingredients that are not developed, imported or registered due to low commercial returns and unfavourable marketing conditions. These drugs are designated for diseases affecting fewer than 1 in 10 000 individuals. Similarly, ODs in Vietnam are described by their availability as pharmaceutical products or active ingredients not developed, imported or registered due to low commercial returns and unfavourable marketing conditions (online supplemental table 6). A summary of ODs definitions is provided based on the country in table 3.

**Table 3:** A summary of ODs definitions is provided based on the country

Country, frequency	# of articles; (%)		(RD) definition	Date
EU/UK (22)	19(20%)	EMA	If the drug is intended for the diagnosis, prevention, or treatment of a life-threatening or chronically and seriously debilitating condition affecting not more than 5 in 10 000 EU people or that it is unlikely that marketing the drug in the EU would generate sufficient benefit for the affected people and for the drug manufacturer to justify the investment.	
		NICE	The current NICE appraisal system means orphan drugs that do not meet HST criteria go through the standard technology appraisal (TA) process, with a cost-effectiveness threshold of ¬£30 k/QALY, or ¬£50 k/QALY when end-of-life criteria are met.	
		EURORDIS	Drugs used in the treatment of rare diseases address significant unmet medical needs and are referred to as orphan drugs because the pharmaceutical industry has little interest under normal market conditions in developing and marketing drugs intended for only a small number of patients suffering from very rare condition.	(2011c)
		The Orphan Medicinal Product Regulation	Defines Orphan Medicinal Products (OMPs) as products for diagnosis, prevention, or treatment of life-threatening or very serious conditions that affect no more than 5 in 10 000 people in the European Union.	
		The Netherlands	Defines orphan drug‚ as either having an official EU orphan designation or if it targets a disease with a prevalence of <1 in 150 000 and shows a clinically proven therapeutic benefit and no other registered medicine exists.	
		Poland	There is no specific formal threshold for orphan designations, there is only a general cost-effectiveness threshold that equals 3× GDP per capita for ICUR/QALY (for CUA) or ICER/LYG (for CEA), which in 2014 is approximately €26 800.	
USA (9)	8(9%)	FDA	The defines an OD as ‘one intended for the treatment, prevention or diagnosis of a rare disease or condition, which is one that affects less than 200 000 persons in the USA’ (which equates to approximately 6 cases per 10 000 population) ‘or meets cost recovery provisions of the act’.	
		Orphan Drug Act (ODA)	Orphan drug on the basis of unprofitability: one intended for the diagnosis, treatment, or prevention of a rare disease or condition in the United States, such that there was no reasonable expectation that the costs of developing the drug would be recovered from its sales in the United States. This definition was amended in 1984 to provide, in addition, a prevalence threshold of 200 000 persons affected by the disease. condition of interest in the United States as a surrogate for the lack of profitability.	
			Orphan product‚ as one that is intended to treat a rare disease or condition that affects fewer than 200 000 people in the United States OR as a product which will not be profitable within seven years of approval by the FDA.	
Korea (2)	2 (2%)	the Orphan Drug Centre	Supplies medicines for diseases affecting fewer than 1 in 20 000.	
		the Korea Ministry of Food and Drug Safety formulates ODs	Drugs used for a disease with 20 000 or fewer patients (population with the disease) and diseases for which adequate treatments or drugs have not yet been developed, or drugs that significantly improve safety or efficacy compared to existing alternatives, are designated as OD.	
China (2)	2 (2%)		Orphan drugs are defined by their availability as pharmaceutical products or active ingredients not developed, imported, or registered owing to low commercial returns and unfavorable marketing conditions.	

CEAcost-effectiveness analysisCUAcost-utility analysisEMAEuropean Medicines AgencyEUEuropean UnionEURORDISEuropean Organisation for Rare DiseasesFDAFood and Drug AdministrationGDPgross deomestic productHSTHighly Specialised TechnologiesICERincremental cost-effectiveness ratioICURincremental cost-utility ratioLYGlife years gainedNICENational Institute for Health and Care ExcellenceODsorphan drugsQALYquality- adjusted life year

### UOD definitions

One study from the UK defined UODs as drugs for diseases with an extremely low prevalence, often less than 0.18 per 10 000 individuals. Three studies introduced the NICE definition for ‘ultra-orphan’ drugs as those targeting conditions with less than 1 case per 50 000 persons. These drugs are typically granted approval for the treatment of diseases that affect fewer than 1000 patients, underscoring their exceptional rarity. In England, the Highly Specialised Technologies Programme has implemented cost-effectiveness thresholds for UODs, while the WHO provides specific recommendations for cost thresholds. Scotland has introduced a distinct definition that places emphasis on conditions affecting fewer than 1 in 50 000 individuals. Furthermore, Scotland has also redefined its criteria for UODs to facilitate early access programmes and streamline reimbursement processes, with a particular focus on conditions impacting approximately 100 individuals. Table 4 provide a summary of UODs definitions based on the country.

**Table 4:** A summary of UODs definitions is provided based on the country

Country, frequency		(UOD) definition	Date
UK	NICE	Drugs with indications for conditions with a prevalence of less than 1 per 50 000 persons	
Scotland	The Scottish government	New definition of ultraorphan medicines that can treat very rare conditions affecting fewer than 1 in 50 000 people—approximately 100 people or fewer in Scotland	
England		HST for ultra-orphan indications Euro113,900–3 41 700/QALY in England	
	WHO	WHO recommends a WTP of <3 times GDP per capita/QALY	
Scotland		New definition for ultra-orphan drugs: medicines that are used to treat a condition with a prevalence of 1 in 50 000 or less or around 100 people in Scotland, which will mostly be used to facilitate early access programs and reimbursement processes	Effective from October 2018

GDPgross deomestic productHSTHighly Specialised TechnologiesNICENational Institute for Health and Care ExcellenceQALYquality-adjusted life yearUODultraorphan drugWTPwillingness to pay

### Qualitative criteria

The review identified 35 qualitative criteria for RDs, 37 for ODs, 7 for URDs and 11 for UODs. The identified qualitative criteria were categorised into seven themes related to RDs, URDs, ODs and UODs: nature, aetiology, disease nature affecting the patients, disease nature affecting the patient’s society, population characteristics, benefits from taking the treatment and indications (online supplemental table 9).

The most frequent qualitative criteria used in defining RDs and URDs were ‘disease’ 148 times and 13 times, respectively, and ‘condition’ 30 times and three times, respectively. For ODs and UODs, the most frequent qualitative criteria were ‘drugs’ 83 times and eight times, respectively, and ‘medical products’ 36 times and two times, respectively. In terms of aetiology, the term ‘genetic’ was used seven times for RDs and once for ODs. Interestingly, ‘hereditary’ was exclusively reported for ODs. The qualitative criterion ‘life-threatening’ was found 23 times and ‘debilitating’ 21 times for RDs, while for ODs, these qualitative criteria appeared 20 and 10 times, respectively. Some qualitative criteria were used to assess the extent of the impact on society, whether the disease was rare or common. The subtheme ‘low prevalence’ appeared 12 times in definitions related to RDs, similarly describing ‘low-occurrence criteria’, ‘infrequent population affliction’, and a ‘small number of patients with RDs’. However, no data pertaining to URDs, ODs or UODs were identified. Notably, the theme ‘benefits from taking the treatment’ was found to be associated only with ODs. In the indications theme, the qualitative criteria ‘treatment and prevention’ were used repeatedly (55 and 23 for ODs and 7 and 1 for RDs, respectively) (online supplemental table 10).

### Quantitative criteria

These quantitative criteria yielded 10 criteria for RDs, five criteria for ODs, four for URDs and three for UODs (online supplemental table 9).

In the context of defining RDs, ODs and their subtypes, quantitative criteria were less common than qualitative criteria. The most popular metric was ‘prevalence’, rather than ‘incidence’, ‘incidence rate’, ‘number of cases’, ‘threshold’, ‘estimated measures’, ‘range’, ‘percentage’, or ‘frequency’. Quantitative criteria such as ‘cost-effective threshold’ and ‘annual budget impact for a particular indication’, as well as ‘willingness-to-pay’, were exclusively recorded for ODs (online supplemental table 11).

## Discussion

This review sheds light on various definitions and criteria used by different countries and stakeholders, provides deeper insights into different elements, promoting the development of strong criteria and facilitates policy dialogue. The present analyses revealed inconsistency in definitions; regional disparities in RD occurrence range from approximately 5000 to 8000^24^; and various terminologies and criteria used to define RDs, ODs and their subtypes.

Some definitions rely on qualitative criteria, such as disease severity, life-threatening or hereditary nature, or the presence of alternative treatment options.^7 25^ These subjective criteria lack substantial evidence and vary based on the specific organisation that uses the term. However, the UK^26^ adopts similar criteria to those used by the EMA to define RDs, suggesting a degree of alignment in the RD classification between Europe and the UK. The European Organisation for Rare Diseases (EURORDIS) definition has a broader scope because it includes both RDs and neglected diseases within the classification of ODs.^27^ This inclusion acknowledges diseases that may be neglected even if they are not strictly rare.

Additionally, we observe that historical differences in definitions have had tangible consequences on healthcare outcomes and drug development priorities over recent decades. For instance, the variation in prevalence thresholds between the USA (fewer than 200 000 individuals) and the EU (fewer than 1 in 2000) has influenced patient eligibility for support and access to treatments, with different thresholds potentially limiting access in regions with more restrictive definitions. These discrepancies have also shaped pharmaceutical investment strategies, as varying definitions impact the perceived market size and economic feasibility of developing treatments for RDs in different regions.

There has been controversy surrounding the term ‘orphan’ in the context of ODs, reflecting differences in interpretations across countries. Initially coined in the early 1960s to describe a class of drugs for RDs, the term highlighted the economic disincentives for developing treatments due to limited profitability. However, by the 1990s, government incentives made RD drug development more viable.^28^ In the UK, the use of the term ‘orphan’ has been criticised, particularly by Rosalind Hurley of the EMA, who expressed regret over its usage.^28^ Despite this criticism, Richter *et al*^7^ argue that the term is consistent in referring to technologies for RDs. In Australia, ODs refer to medicines, vaccines or in vivo diagnostic agents used to treat, prevent or diagnose or not available to treat, prevent or diagnose another disease.^29^ This provides a broader understanding of the term and its application in different regions.

Disease severity is considered a critical criterion in evaluating the impact of ODs on health-related outcomes in patients, considering that diseases can substantially affect both health and health-related quality of life.^30^ Haendal *et al* ^31^ recommended that a multitude of overlapping terminologies, models and metadata exist for the identification and classification of RDs. Failure to do so can have substantial consequences, affecting drug approvals, market entry prices and reimbursement recommendations and ultimately impeding patient access to ODs.

Additionally, some definitions depend on quantitative criteria, such as the disease prevalence threshold, which constitutes the favoured epidemiological element used in 58% of RD definitions.^7^ However, establishing a prevalence threshold poses challenges due to diverse information sources. This challenge is exacerbated by the absence of firmly established diagnostic criteria or coding systems necessary to gather these data.^32^ As a result, certain diseases could be deemed rare in one country but not in another owing to genetic population diversity, environmental or societal pressures and variations in survival challenges across different regions.^10^

One study^7^ presented a comprehensive overview of RD definitions worldwide, collating 296 definitions from 1109 organisations across 32 international jurisdictions. The findings indicated the common use of terms such as ‘RDs’ and ‘ODs,’ while descriptive qualifiers such as ‘life-threatening’ were less prevalent. Moreover, 88% of the investigations specified prevalence thresholds ranging from 5 to 76 cases per 100 000 people, with 66% of jurisdictions adopting thresholds between 40 and 50 cases per 100 000 individuals. The study^7^ underscored the substantial diversity in defining RDs across various jurisdictions and organisational structures. This highlights the necessity for standardisation, particularly in objective criteria such as prevalence thresholds, while recommending the avoidance of subjective qualifiers to achieve a harmonised definition of RDs. Despite the widespread use of terms such as ‘RDs’ and ‘ODs’, the study emphasised the importance of focusing on standardised metrics to ensure clarity and consistency in identifying RDs globally.

This SLR emphasises the importance of developing a local definition for each country, regardless of the criteria applied. Subjective qualifiers can occasionally provide additional context or complexity to the description of RDs, ODs and their subtypes. However, relying too heavily on subjective standards may lead to inconsistent results and implementation challenges. For comprehensive definitions of RDs, ODs and their subtypes, it is better to combine qualitative and quantitative criteria, which should be reviewed and updated periodically.

Additionally, differences in disease classification across regions can lead to significant disparities in patient care, research funding and access to treatments. For instance, cystic fibrosis^33^ is classified as rare in Europe and North America, where it benefits from OD designations, incentivising pharmaceutical companies to develop treatments. However, in regions where it is less common, the lack of this classification can limit research initiatives and access to specialised care.^34^ Similarly, sickle cell anaemia is considered rare in the USA^35^ and UK^35^ but is more common in parts of Africa,^36^ the Middle East,^36^ eastern and southwestern regions of SA,^35^ where healthcare systems are better equipped to handle it. In contrast, in countries where sickle cell is classified as rare, patients may face limited treatment options and fewer specialists.^37^ These examples highlight how the classification of a disease as rare in one country and common in another can lead to inconsistencies in care, treatment availability and research focus, underscoring the importance of harmonising definitions across regions.

In summary, an exploration of the worldwide definitions of RDs, ODs and their subtypes provides a comprehensive understanding of their complex nature. The diversity in criteria among nations and institutions accentuates the problem of defining them, influenced by genetic variations, societal factors and regional disparities. This important fact illuminates the critical challenges and factors required to address these conditions and advance the development of treatments for individuals affected by RDs globally.

### Recommendations for future use

This study highlights the importance of establishing a country-specific consensus on the definition of the distinctive combination of genetic, phenotypic and environmental characteristics as well as sociocultural and economic factors. RDs should be linked to individuals to steer the research and enhance the diagnosis and care of patients with RDs and the availability of treatments^38^ based on scientific principles. Qualitative and quantitative criteria and subthemes should be included in the definition. Therefore, understanding the economic and ethical principles of and healthcare burdens associated with RDs, ODs and their subtypes is essential for policy-makers to shape policies, especially in underdeveloped policy areas. Moreover, there is a need for international collaboration and data exchange to improve the global understanding and treatment of RDs, which in turn can affect pricing, reimbursement and patient access to ODs. Additionally, more robust evidence is needed to effectively implement the United Nations 2030 Agenda principles and Sustainable Development Goals of ‘leaving no one behind’, ‘reducing inequalities’ and ‘addressing the needs of those furthest behind first*’* to support the RD community.

## Conclusion

A comprehensive study on RD, OD and subtype definitions across countries is lacking. In particular, these definitions are considered outdated, with no scientific grounding. There is a need to address problems associated with diseases that impact only a small percentage of the population. These definitions are meant to provide a framework for identifying and supporting the development of ODs. Therefore, local evaluations of qualitative and/or quantitative criteria are needed to shift therapeutic outcomes from treatment to transformative and curative treatment, to gather comprehensive patient data, to accurately determine disease prevalence, and to ensure equity and equality in accessing appropriate treatments. It is imperative for each country to develop a local definition or reporting system or establish a national registration programme. This approach would not only facilitate the collection of vital health information but also foster a more effective healthcare ecosystem that addresses the needs of individuals affected by these conditions.

## supplementary material

10.1136/bmjopen-2024-086527 online supplemental file 1

10.1136/bmjopen-2024-086527 online supplemental file 2

10.1136/bmjopen-2024-086527 online supplemental file 3

10.1136/bmjopen-2024-086527 online supplemental file 4

10.1136/bmjopen-2024-086527 online supplemental file 5

10.1136/bmjopen-2024-086527 online supplemental file 6

## Data Availability

All data relevant to the study are included in the article or uploaded as supplementary information.
