# Supplementary material for: Global insight into rare disease and orphan drug definitions: a systematic literature review
Source: BMJ Open. 2025 Jan 25;15(1):e086527. doi: 10.1136/bmjopen-2024-086527 (PMC11784410; doi:10.1136/bmjopen-2024-086527)
Supplement: online supplemental file 1 [file bmjopen-15-1-s001.pdf]

Supplementary Table 1: Research question: What are the criteria to define Rare Diseases and Orphan Drugs globally?

Concept 1: Criteria / Concept 2: Define/ Concept 3: Rare Disease(s)/ Concept 4: Orphan Drug(s)

	Concept 1	Concept 2	Concept 3	Concept 4	Total	limit to english & human
PubMed	Criteria [All Fields] OR Standard*[All Fields] OR classification [All Fields] OR Measure*[All Fields] OR Condition*[All Fields] OR Principle*[All Fields] OR Requirement*[All Fields] OR Scale*[All Fields] OR Parameter*[All Fields] OR Indicator*[All Fields] OR Norm*[All Fields]	Defin*[All Fields] OR Mean*[All Fields] OR Description [All Fields] OR Character*[All Fields] OR Explan*[All Fields] OR delineate [All Fields] OR detail [All Fields] OR interpret[All Fields] OR determine[All Fields] OR elucidate[All Fields] OR illustrate[All Fields] OR exemplify[All Fields]	"Rare Diseases"[Mesh] OR “Orphan disease*”[All Fields] OR “Rare condition*”[All Fields] OR “Rare disorder*”[All Fields] OR “Rare disability*”[All Fields] OR “Neglected disease*”[All Fields] OR “Undiagnosed disease*”[All Fields] OR “Low-frequency disease*”[All Fields] OR “life-threatening disease*”[All Fields] OR “debilitating disease*”[All Fields] OR “severe disease*”[All Fields] OR “intractable disease*”[All Fields]	"Orphan Drug Production"[Mesh] OR “Orphan medicinal product*” [All Fields] OR “Orphan product*”[All Fields] OR “Orphan subset*”[All Fields] OR “Orphan indication*”[All Fields] OR “Highly specialized technolog*”[All Fields] OR “Priority review drug*”[All Fields] OR "Orphan Drug*"[All Fields]	(((Criteria [All Fields] OR Standard*[All Fields] OR classification [All Fields] OR Measure*[All Fields] OR Condition*[All Fields] OR Principle*[All Fields] OR Requirement*[All Fields] OR Scale*[All Fields] OR Parameter*[All Fields] OR Indicator*[All Fields] OR Norm*[All Fields]) OR (Defin*[All Fields] OR Mean*[All Fields] OR Description [All Fields] OR Character*[All Fields] OR Explan*[All Fields] OR delineate [All Fields] OR detail [All Fields] OR interpret[All Fields] OR determine[All Fields] OR elucidate[All Fields] OR illustrate[All Fields] OR exemplify[All Fields])) AND ("Rare Diseases"[Mesh] OR "Orphan disease*"[All Fields] OR "Rare condition*"[All Fields] OR "Rare disorder*"[All Fields] OR "Rare disability*"[All Fields] OR "Neglected disease*"[All Fields] OR "Undiagnosed disease*"[All Fields] OR "Low-frequency disease*"[All Fields] OR "life-threatening disease*"[All Fields] OR "debilitating disease*"[All Fields] OR "severe disease*"[All Fields] OR "intractable disease*"[All Fields])) AND ("Orphan Drug Production"[Mesh] OR "Orphan medicinal product*" [All Fields] OR "Orphan product*"[All Fields] OR "Orphan subset*"[All Fields] OR "Orphan indication*"[All Fields] OR "Highly specialized technolog*"[All Fields] OR "Priority review drug*"[All Fields] OR "Orphan Drug*"[All Fields])	
	11,155,322	14,855,618	78,992	2,409	435	334
Medline	(Criteria or Standard* or classification or Measure* or Condition* or Principle* or Requirement* or Scale* or Parameter* or Indicator* or Norm*).mp. [mp=title, abstract, original title, name of substance word, subject heading word, floating sub-heading word, keyword heading word, organism supplementary concept word, protocol supplementary concept word, rare disease supplementary concept word, unique identifier, synonyms]	(Defin* or Mean* or Description or Character* or Explan* or delineate or detail or interpret or determine or elucidate or illustrate or exemplify).mp. [mp=title, abstract, original title, name of substance word, subject heading word, floating sub-heading word, keyword heading word, organism supplementary concept word, protocol supplementary concept word, rare disease supplementary concept word, unique identifier, synonyms]	(Orphan disease* or Rare condition* or Rare disorder* or Rare disability* or Neglected disease* or Undiagnosed disease* or Low-frequency disease* or life-threatening disease* or debilitating disease* or severe disease* or intractable disease* or Rare Disease*).mp. [mp=title, abstract, original title, name of substance word, subject heading word, floating sub-heading word, keyword heading word, organism supplementary concept word, protocol supplementary concept word, rare disease supplementary concept word, unique identifier, synonyms]	(Orphan medicinal product* or Orphan product* or Orphan subset* or Orphan indication* or Highly specialized technolog* or Priority review drug* or Orphan Drug* or Orphan Drug Production*).mp. [mp=title, abstract, original title, name of substance word, subject heading word, floating sub-heading word, keyword heading word, organism supplementary concept word, protocol supplementary concept word, rare disease supplementary concept word, unique identifier, synonyms]	1 OR 2 And 3 and 4	
	10,653,511	7,966,623	98,302	2,236	510	334
Embase	(Criteria or Standard* or classification or Measure* or Condition* or Principle* or Requirement* or Scale* or Parameter* or Indicator* or Norm*).mp. [mp=title, abstract, heading word, drug trade name, original title, device	(Defin* or Mean* or Description or Character* or Explan* or delineate or detail or interpret or determine or elucidate or illustrate or exemplify).mp. [mp=title, abstract, heading word, drug trade name, original title, device manufacturer, drug manufacturer, device trade	(Orphan disease* or Rare condition* or Rare disorder* or Rare disability* or Neglected disease* or Undiagnosed disease* or Low-frequency disease* or life-threatening disease* or debilitating disease* or severe disease* or intractable disease* or Rare	(Orphan medicinal product* or Orphan product* or Orphan subset* or Orphan indication* or Highly specialized technolog* or Priority review drug* or Orphan Drug* or Orphan Drug Production*).mp. [mp=title, abstract, heading word, drug	1 OR 2 And 3 and 4	

	manufacturer, drug manufacturer, device trade name, keyword, floating subheading word, candidate term word]	name, keyword, floating subheading word, candidate term word]	Disease*).mp. [mp=title, abstract, heading word, drug trade name, original title, device manufacturer, drug manufacturer, device trade name, keyword, floating subheading word, candidate term word]	trade name, original title, device manufacturer, drug manufacturer, device trade name, keyword, floating subheading word, candidate term word]		
	13,859,313	10,574,947	160,442	4828	1,010	760
**Scopus**	TITLE-ABS-KEY (criteria OR standard* OR classification OR measure* OR condition* OR principle* OR requirement* OR scale* OR parameter* OR indicator* OR norm*)	TITLE-ABS-KEY (defin* OR mean* OR description OR character* OR explan* OR delineate OR detail OR interpret OR determine OR elucidate OR illustrate OR exemplify)	TITLE-ABS-KEY ("Orphan disease*" OR "Rare condition*" OR "Rare disorder*" OR "Rare disability*" OR "Neglected disease*" OR "Undiagnosed disease*" OR "Low-frequency disease*" OR "life-threatening disease*" OR "debilitating disease*" OR "severe disease*" OR "intractable disease*" OR "Rare Disease*")	TITLE-ABS-KEY ("Orphan medicinal product*" OR "Orphan product*" OR "Orphan subset*" OR "Orphan indication*" OR "Highly specialized technolog*" OR "Priority review drug*" OR "Orphan Drug Production*" OR "Orphan Drug*")	(TITLE-ABS-KEY (criteria OR standard* OR classification OR measure* OR condition* OR principle* OR requirement* OR scale* OR parameter* OR indicator* OR norm*)) OR (TITLE-ABS-KEY (defin* OR mean* OR description OR character* OR explan* OR delineate OR detail OR interpret OR determine OR elucidate OR illustrate OR exemplify)) AND (TITLE-ABS-KEY ("Orphan disease*" OR "Rare condition*" OR "Rare disorder*" OR "Rare disability*" OR "Neglected disease*" OR "Undiagnosed disease*" OR "Low-frequency disease*" OR "life-threatening disease*" OR "debilitating disease*" OR "severe disease*" OR "intractable disease*" OR "Rare Disease*")) AND (TITLE-ABS-KEY ("Orphan medicinal product*" OR "Orphan product*" OR "Orphan subset*" OR "Orphan indication*" OR "Highly specialized technolog*" OR "Priority review drug*" OR "Orphan Drug Production*"))	
	29,871,274	21,496,075	134,422	4,160	782	667
**WOS**	ALL FIELDS: (criteria OR standard* OR classification OR measure* OR condition* OR principle* OR requirement* OR scale* OR parameter* OR indicator* OR norm*) Timespan: All years. Indexes: SCI-EXPANDED, SSCI, A&HCI, CPCI-S, CPCI-SSH, ESCI., CPCI-S, CPCI-SSH, ESCI.	ALL FIELDS: (defin* OR mean* OR description OR character* OR explan* OR delineate OR detail OR interpret OR determine OR elucidate OR illustrate OR exemplify) Timespan: All years. Indexes: SCI-EXPANDED, SSCI, A&HCI, CPCI-S, CPCI-SSH, ESCI.	ALL FIELDS: ("Orphan disease*" OR “Rare condition*” OR “Rare disorder*” OR “Rare disability*” OR “Neglected disease*” OR “Undiagnosed disease*” OR “Low-frequency disease*” OR “life-threatening disease*” OR “debilitating disease*” OR “severe disease*” OR “intractable disease*” OR “Rare Disease*") Timespan: All years. Indexes: SCI-EXPANDED, SSCI, A&HCI, CPCI-S, CPCI-SSH, ESCI.	ALL FIELDS: ("Orphan medicinal product*" OR "Orphan product*" OR "Orphan subset*" OR "Orphan indication*" OR "Highly specialized technolog*" OR "Priority review drug*" OR "Orphan Drug Production*" OR "Orphan Drug*") Timespan: All years. Indexes: SCI-EXPANDED, SSCI, A&HCI, CPCI-S, CPCI-SSH, ESCI.	#7 AND #6 AND #5 Timespan: All years. Indexes: SCI-EXPANDED, SSCI, A&HCI, CPCI-S, CPCI-SSH, ESCI. ...Less	
	20,665,577	18,096,480	90,196	3,462	646	617
					Totla	2,712
