# Supplementary material for: Global insight into rare disease and orphan drug definitions: a systematic literature review
Source: BMJ Open. 2025 Jan 25;15(1):e086527. doi: 10.1136/bmjopen-2024-086527 (PMC11784410; doi:10.1136/bmjopen-2024-086527)
Supplement: online supplemental file 3 [file bmjopen-15-1-s003.pdf]

Supplementary Table 3: List of included studies

Year	Country/ Jurisdiction / Organization	Study design	Aim	Definition			
				RD	OD	URD	UOD
1992[18]	USFAD/ Orphan Drug Act, P.L. 97-414, 1983.	Review	This paper examines some of the special problems that are associated with the design and implementation of studies to evaluate the safety and efficacy of orphan drugs.	The legal definition of a rare disease or condition is one that "either (A) affects less than 200,000 persons in the United States, or (B) affects more than 200,000 in the United States and for which there is no reasonable expectation than the cost of developing and making available in the United States a drug for such disease or condition will be recovered from sales in the United States of such drug.	Orphan drug and biological products are Pharmaceuticals that are generally not considered to be attractive for commercial development. Generally, orphan drug products are used in treating or preventing rare diseases.		
2002[19]	United States	Book - Chapter	The information presented is directed both at the fortunate individuals al-ready involved in drug development and at those adventuresome sorts who are considering entering the field. We hope this book will provide readers with in-sights into this exciting arena and begin to explain the complicated process of developing a promising new drug		Orphan products are used to treat rare diseases or conditions that by definition, affect fewer than 200,000 people (or up to 1 in 1300) in the United States.		
2003[20]	United States; Paris, France/ European Medicinal Evaluation Agency	Review	To analyse the American and European experience on the Orphan Medicinal Products.		A medical product can receive the designation as an orphan medical product if it can be established that it is intended for the diagnosis, prevention, or treatment of a life-threatening or chronically debilitating condition affecting not more than 5 in 10 thousand persons in the EU. American definition of OD not clear		
2004[21]	United States; India, Japan, Australia/ US FDA	Review	This article reviews the bias for classification of orphan drugs, the discovery of orphan drugs, and attempts by pharmaceutical industries, academician (scientist) and practicing physician, with their respective perspectives, advantages and disadvantages in discovery and development of orphan drugs and some historical aspects.	Rare disease or condition is any disease or condition which affects less than two hundred thousand persons in the United States or affects more than two hundred thousand persons in the United States, but for which there is no reasonable expectation that the cost of developing and making available, a drug for such disease or condition will be recovered from its sales in US.	- Orphan Drugs have been defined in USA as those drugs intended to treat either a rare disease or more common disease where the sponsor cannot make any profit.<br>- As per the definition US FDA, Orphan drugs are those drugs used in diseases or circumstances which occur so infrequently in USA, that there is no reasonable expectation that the cost of developing and making available, a drug for such disease or condition will be recovered from its sales in the USA.<br>- The availability of orphan drugs to patients before being granted a Marketing Authorization is possible. USFDA designated orphan drug with t-IND (testament Investigational New Drug) in some cases such as when the drug is intended for the treatment of a serious or life-threatening disease, when no alternative drug or treatment is available, and thirdly, the product is in the process of clinical trials and in an active phase of Marketing Authorization application		
2005[22]	UK, United States, Japan, Australia	Education and debate	We examine the justifications for special status for rare diseases and ask whether the cost effectiveness of drugs for rare or very rare diseases should be treated differently from that of other drugs and intervention.	Definitions of orphan disease: United States diseases with a prevalence of 7.5/10 000; Japan diseases with a prevalence of 4.0/10 000; Australia diseases with a prevalence of 1.1/10 000; and EU diseases with a prevalence of 5.0/10 000.			The UK defines Ultra Orphan Drug define as drug for diseases with a prevalence of 0.18/10 000 or less
2006[23]	European Union Regulation (EC) No 141/2000	Book - Chapter		Rare diseases, including those of genetic origin, are life-threatening or chronically debilitating diseases which are of such low prevalence that special combined efforts are needed to address them so as to prevent significant morbidity or perinatal or early mortality or a considerable reduction in an individual's quality of life or socio-economic potential. As a guide, low prevalence is taken as prevalence of less than 5 per 10,000 persons in the European Union [1]"	-The lack of drug development for products intended for the prevention, treatment or diagnosis of rare diseases has made necessary the creation of a number of incentives to stimulate the development of such products. These drugs are known as orphan drugs.<br>- In the EU a medicinal product to treat rare diseases is designated as an orphan medicinal product based on either a demonstrated insufficient return on investment or the rarity of the condition and, the absence of satisfactory method of diagnosis, prevention or treatment of the condition concerned is authorized, or, if such method exists, the assumption that the product		

Year	Country/ Jurisdiction / Organization	Study design	Aim	Definition			
				RD	OD	URD	UOD
					will be of significant benefit to those affected by the condition. -Criteria for orphan designation are the following: Firstly, a criterion is based on the low prevalence ("rarity") of the condition, i.e., condition affecting not more than 5 in 10,000 persons in the European Union. Alternatively, the sponsor can apply for more frequent conditions if it can be shown that the development would not be covered by sufficient financial return, i.e., if without incentives it is unlikely that the marketing of the medicinal product in the Community would generate sufficient return to justify the investment by the sponsor. Secondly, it is necessary for designation that the life-threatening or debilitating nature of the condition is justified. The sponsor is invited to provide any scientific and/or medical references that may support the life-threatening or seriously debilitating nature of the condition. Finally, the sponsors are also required to demonstrate that either there exists no satisfactory method of diagnosis, prevention, or treatment of the condition in question or, if such methods exist, that the medicinal product will be of significant benefit to those affected by that condition		
2006[24]	USA Orphan Drug Act, European	Policy And Practice	In this paper we propose selection criteria for an Orphan Medicines Model List that could form a departure point for future work towards an extensive WHO Orphan Medicines Programme.	In the USA Orphan Drug Act, the definition relates to an absolute number (<200 000 patients in the USA), while the European regulation uses a relative measure (<5 cases per 10 000 inhabitants) and requires disorders to be life threatening and/or chronically debilitating.			
2008[25]	United States	Book - Chapter		The legislative definition for a rare disease in the United States is one with a prevalence of less than 200,000 persons or, if over 200,000 persons, one for which there is no reasonable expectation of recovering drug development costs within seven years of market approval			
2009[26]	United States of America, Japan, EU, Australia, and Taiwan	Review		A rare disease is defined as a disease or condition affecting fewer than 200,000 persons in the United States of America. <50,000 patients in Japan, The EU defines rare diseases as life threatening or chronically debilitating diseases which are of such low prevalence in 2,000) that special combined efforts are needed to address them. Australia: < 2000 individuals. Taiwan: < 1 in 10,000 people.			
2010[27]	United States/ Orphan Drug Act of 1983	Book	- To provide a convenient repository for the substantial work that has been accomplished by individual investigators treating rare genetic disorders with simple molecules.<br>- To provide a handbook that will enable potential clinician/ scientists and others to rapidly survey the field, thus ascertaining what has been done and what can yet be done.	In that legislation, an orphan disease was defined as a condition that affects fewer than 200,000 Americans." Serious, life-threatening disorders across the age span.	Serious, life-threatening disorders across the age span.		
2010[28]	United States/ Orphan Drug Act	Review			The Act initially defined an orphan drug on the basis of unprofitability: one intended for the diagnosis, treatment, or prevention of a rare disease or condition in the United States, such that there was no reasonable expectation that the costs of developing the drug would be recovered from its sales in the United States. This definition was amended in 1984 to provide, in addition, a prevalence threshold of 200,000 persons affected by the disease or condition of interest in the United States as a surrogate for the lack of profitability.		
2010[29]	United States/ the Office of Rare Diseases Research (ORDR)	Book- Chapter	This chapter will focus on many of the activities of the ORDR and include other significant activities related to rare diseases research and orphan products development	The disorders and conditions in the rare diseases category are defined by the prevalence figure of fewer than 200,000 people in the United States with the specific disease. An estimated 25			

Year	Country/ Jurisdiction / Organization	Study design	Aim	Definition			
				RD	OD	URD	UOD
				million to 30 million people in the United States have a rare disease or condition."			
2010[30]	UK; EU, World Health Organisation, Australia, Japan and the United States	Book-Chapter		-Rare diseases, including those of genetic origin, are defined by the European Union as life-threatening or chronically debilitating diseases which are of such low prevalence (less than 5 per 10,000) that special combined efforts are needed to address them so as to prevent significant morbidity or perinatal or early mortality or a considerable reduction in an individual, quality of life or socio-economic potential.<br>-According to the World Health Organisation, a rare disease affects at most 6.5 out of every 10,000 individuals.<br>-Australia, Japan, and the United States have set prevalence's of 1.16, 4.07 and 6.68 per 100,000 individuals respectively for a given rare disease."			
2010[31]	United States/ The Orphan Drug Act	Review			The Orphan Drug Act defined an ‚orphan product, as one that is intended to treat a rare disease or condition that affects fewer than 200,000 people in the United States OR as a product which will not be profitable within seven years of approval by the FDA. There are over 6,000 conditions that meet the definition of a rare disease.		
2011[32]	UK, WHO, US FDA, EU, Japan, Australia:	General review	This article aims to provide a description of principal aspects of policy and practice associated with orphan drugs and treatments of rare diseases and give perspectives for 2011 on new and emerging approaches for addressing patient access."<br>"This article summarizes the current state of international orphan drug patient access and describes developments up to 2011. Emerging policies and practices that will affect patient access in 2011 and beyond are also explored."	-WHO: Frequency of 6.5-10/ 10,000 inhabitants US FDA: Affecting, <7 patients/10,000 residents (estimated to affect about 200,000 patients/year<br>-EU: Affecting ≤ 5 patients/10,000 residents (estimated to affect about 30 million EU citizens)<br>-Japan: Affecting <40/100,000 of the population.<br>-Australia: Affecting <11/100,000 inhabitants or ≤2000Australians	Drugs used in the treatment of rare diseases address significant unmet medical needs and are referred to as orphan drugs because, as described by EURORDIS (2011c) , the pharmaceutical industry has little interest under normal market conditions in developing and marketing drugs intended for only a small number of patients suffering from very rare condition.	Ultra-orphan diseases, in the UK, the term refers to chronic diseases with a prevalence of 1 in 50,000 of the population	
2011[33]	Spain	Abstract	We assessed the characteristics and outcomes of the new drug development for rare diseases in the EU.		In the European Union (EU), orphan drugs are used for the diagnosis, prevention, or treatment of life-threatening or serious conditions that affect5 in 10,000 people (NOTE THE OVERLAP BETWEEN ORPHAN DRUG AND RARE DISEASE DEFINITION)		
2011[34]	Canada	Abstract	The scope of this study is to describe the ODs regulations in Canada, evidence requirements by the national regulatory agency, national and regional funding criteria, market access challenges associated with ODs, and approaches to obtain access to ODs in Canada.	The Canadian Organization of Rare Diseases (CORD) defines a rare disease as one that afflicts less that 1 person in 200 000.			
2012[35]	Middle East (Egypt, Iran, Turkey, Iraq, Saudi Arabia, Yemen, Syria, United Arab Emirates or UAE, Israel, Jordan, Lebanon, Oman, Kuwait, Qatar, Bahrain, and Cyprus) plus the Palestinian territories of the West Bank and the Gaza Strip	Policy Forum			An orphan drug is a drug developed specifically to treat a rare medical condition		
2012[36]	United States	Editorial		-The terms‚ orphan diseases‚ and‚ rare diseases‚ are commonly used interchangeably worldwide and have been defined as ‚any disease or condition that affects a small percentage of the population.<br>-The US Rare Diseases Act of 2002 defines rare disease strictly according to prevalence, as does Japan.			

Year	Country/ Jurisdiction / Organization	Study design	Aim	Definition			
				RD	OD	URD	UOD
				-The European Commission on Public Health defines rare diseases as ‚life-threatening or chronically debilitating diseases which are of such low prevalence that special combined efforts are needed to address them.<br>-The definition of ‚low prevalence‚ varies between countries but usually ranges from 1/1,000 to 1/200,000<br>-The alternative term‚ orphan disease‚ is used in reference to a combination of the paucity of treatment availability, lack of resources, and severity of disease.			
2012[37]	United States	Review	- In this article we present the findings of this analysis, which, consistent with the IOM recommendation, are intended to identify factors correlating with rare disease product approvals that could inform future development programs, and to identify areas where additional resources might be directed.<br>- In this work we provide an up-to date analysis of drug, target interactions for approved and clinical trial drugs and examine the major developments and trends in pharmaceutical development<br>- For the purpose of supporting rare disease product development, we undertook an evaluation of CDER‚ rare disease marketing application history, focusing on a recent five-year period (2006 - 2010).	Rare diseases, which are disorders affecting less than 200,000 persons in the USA, also have considerable unmet medical needs.			
2012[38]	European Union countries	Review	The aim of this study was to quantify both the sales and volume uptake of orphan drugs in Europe and to assess whether a country‚ gross domestic product (GDP) and/or health technology assessment (HTA) influences the orphan drugs‚ market uptake.	In the European Union, a rare disease is defined as a life-threatening or chronically debilitating disease with the prevalence among 50 per 100 000 people or less	Orphan drugs are drugs intended for the treatment of rare diseases.		
2012[39]	Singapore, Taiwan, Korea, and China	Meeting Abstract		-Since 1991, Singapore‚ Orphan Drugs Policy allows patients with life-threatening and severely debilitating diseases with no other treatment options to access approved drugs prescribed by their practitioner.<br>-The Taiwan Foundation for Rare Disorders helped secure the Rare Disease and Orphan Drugs Act in 2000. Diseases affecting fewer than 1 in 10,000 that are officially recognized are eligible for medical coverage.<br>-In Korea, the Orphan Drug Centre supplies medicines for diseases affecting fewer than 1 in 20,000.<br>-In China, in 2011, medical professionals called for legislation to support healthcare, research, orphan drug development, and epidemiological studies for diseases affecting fewer than 1 in 10,000	-Since 1991, Singapore‚ Orphan Drugs Policy allows patients with life-threatening and severely debilitating diseases with no other treatment options to access approved drugs prescribed by their practitioner.<br>-In Korea, the Orphan Drug Centre supplies medicines for diseases affecting fewer than 1 in 20,000.		
2013[40]	Middle East	Critical Review	We provide a critical review of the literature on the availability of orphan drugs in the Middle East.		An orphan drug is a drug developed specifically to treat a rare medical condition.		
2013[41]	United States; UK; and EU	Review	We examined the characteristics of orphan drug (OD) designations and approvals by the US Food and Drug Administration (FDA) and the European Medicines Agency (EMA) between 2000 and 2011.		Criteria for Orphan designation is generally based on the number of patients affected by the disease (<200,000 US patients and <5 in 10,000 EU patients). The EU also requires that a satisfactory alternative treatment is not available or that the new drug is significantly better than drugs currently marketed.		
2013[42]	UK	Conference	- The presentation provides a brief review of all supportive incentives in the field of orphan medicinal products as: the European orphan medicinal product (OMP) regulation, Guideline on Clinical Trials in Small Populations and Commission Regulation (EC) No 2049/2005 / support of small and medium enterprises (SMEs)."<br>- It also introduces the concept of Clinical added value of orphan medicinal products, as one of the key instruments to increase the availability of orphan medicinal products in the member states."		- The orphan drug intended for diagnosis, prevention or treatment of a life threatening or chronic debilitating condition.<br>- The prevalence of the condition, for which the OMP (orphan medicinal product) is intended, must be less than 5 in 10,000"<br>- OMP has to fulfil following criteria:<br>1. Seriousness of the condition the investigated drug must be intended for diagnosis, prevention, or treatment of a life-threatening or chronic debilitating condition.		

Year	Country/ Jurisdiction / Organization	Study design	Aim	Definition			
				RD	OD	URD	UOD
					2. Low prevalence/irretrievable investment the prevalence of the condition, for which the OMP is intended, must be less than 5 in 10,000 or the investigated OMP must be unlikely to generate sufficient return to justify the investment. In some situations, the condition is defined as a subset of another frequent condition. To accept the subset, it is needed to prove that the subset is medically recognizable and the investigated OMP will be effective only in this subset and not in the condition per se.<br>3. Medical need No other treatment is authorised in EU for this condition or if there is one, the designated OMP must provide a significant benefit over the existing method. The significant benefit is given on the basis of/upon clinically relevant advantage or major contribution to patient care EC/847/200		
2013[43]	Taiwan, and Republic of China	Registry data analysis	- This paper aims to describe the prevalence of RDs over time from 2002 to 2011 based on the national RDs registry data in Taiwan".<br>- To describe a general demographic picture of patients with rare diseases in Taiwan and particularly focuses on the prevalence of rare diseases over time, age, and gender distributions.	- Rare disease as a disease whose prevalence is less than 1 in 10,000 in Taiwan.<br>- Taiwan officially included RDs as one type of disability and initiated the RDs disability registry in the social welfare system in 2002 (the Physically and Mentally Disabled Citizens Protection Act, 2001)			
2013[3]	China	Review	In this article, the primary tasks faced by China have been proposed: to call on the government to legislate as soon as possible; to establish information platform of rare diseases and orphan drugs for sharing the global rare diseases resources; to establish Rare Disease Outpatient Service (RDOPS)for improving the level of diagnosis and treatment; to carry out tertiary prevention of the rare diseases; to establish the rare diseases epidemiological surveillance system in our country	- World Health Organization (WHO) defines a rare disease as affecting 65/100 000~100/100 000 persons. A disease is considered as rare when it affects 1 person per 2,000 in Europe, <200 000 people in the United States, <50 000 people (1 person per 2500) in Japan and 1 person per 10 000 in Taiwan. In China, the Chinese Society of Genetic Medicine defines rare disease as 'diseases affect less than one over 500 000 and genetic disorders affect with less than one over 50 000 of the incidences in new-born babies.<br>- Rare diseases are serious chronic diseases, difficulties in obtaining timely, accurate diagnoses and are often life-threatening	Orphan drugs are those intended to diagnose, prevent, or treat rare diseases or pathologies that are serious or life threatening, and whose development costs are superior to the expected return on investment		
2013[44]	Seven European countries, Belgium	Review	This study aimed to identify, describe, and classify MEAs applied to orphan medicinal products (OMPs) by national payers and to analyse their practice in Europe.<br>The present study, focusing on seven European countries, had three main objectives, namely to: (i) examine the processes through which MEAs are implemented by national healthcare payers, (ii) identify, describe, and classify MEAs applied to OMPs by national healthcare payers, and (iii) analyse and compare identified MEAs related to OMPs within and between countries.	Life-threatening or chronically debilitating diseases with a prevalence of 5 out of 10,000 or less			
2013[45]	United States/ Orphan Drug Act (ODA)	Book - Chapter		- Rare diseases, also referred to as orphan diseases, are defined in the United States (US) by the Orphan Drug Act (ODA) as diseases or conditions that affect fewer than 200,000 persons in the US.<br>- Most rare diseases are serious, life-limiting, or life-threatening conditions	Orphan designated drugs are those that are: intended to treat, prevent, or diagnose diseases or conditions affecting fewer than 200,000 persons in the US; and have shown promise, based on supporting evidence, in the treatment of the disease or condition.		
2013[46]	Netherlands	Research Article	In the Netherlands, we decided to build a registry for patients with metabolic disorders and also to optimize the codes for national use in medical and clinical genetics. With these purposes in mind, we developed, with a dedicated group of clinical specialists, a clinically oriented annotation system for metabolic disorders based on two existing national coding systems.	Rare diseases are life threatening or chronically debilitating dis-eases with a prevalence of up to five per 10,000 inhabitants in the European Union (EU)			

Year	Country/ Jurisdiction / Organization	Study design	Aim	Definition			
				RD	OD	URD	UOD
2013[47]	China, WHO, United States, Japan, and Australia	Commentary		- A rare disease is referred to as any disease that affects an extremely small percentage of the population.<br>- The World Health Organization (WHO) defines a disease as a rare disease when its incidence ranges approximately from 0.65-1% in the whole population.<br>- Rare disease is identified in the United States (US), Japan, and Australia when it afflicts less than 200,000 (approx. 0.75% of the population), 50,000 (approx. 0.4% of the population), and 2,000 (approx. 0.1% of the population) people, respectively.<br>- Expert consensus indicates that a rare disease could be identified in China when the incidence of the disease in adults or neonates is less than 1 in 500,000 and 1 in 10,000, respectively.			
2014[48]	Poland	Abstract	The aim of this study was to identify the cost-effectiveness threshold for an orphan designation in Poland.		- According to criteria specified by the European Medicines Agency (EMA) a medicine must meet a strict criteria to qualify for orphan designation, such as: treatment, prevention or diagnosis of a disease that is life-threatening or chronically debilitating; disease prevalence level in the European Union (EU) of no more than 5 cases in 10,000 patients is necessary no satisfactory method of disease diagnosis, prevention, treatment or if such method exists, the drug must deliver significant benefits to patients.<br>- In Poland there is no specific formal threshold for orphan designations, there is only a general cost-effectiveness threshold that equals 3 x GDP per capita for ICUR/QALY (for CUA) or ICER/LYG (for CEA), which in 2014 is approximately € 26 800.		
2014[49]	UK, US	Review	We aim to highlight how the emergence of omics technologies and the development of integrated , systems medicine, approaches might offer ways to overcome research challenges in rare disease and allow patients to ultimately reap the benefits of better scientific understanding of their condition.	Rare diseases are defined in the European Union as those with a prevalence of < 5 in 10,000 and in the US as diseases that affect fewer than 200,000 US citizens			
2014[50]	Latvia	Conferences	This study aims to determine the trends in reimbursement of ODs in Latvia within the framework of individual reimbursement system in 2008, 2011.	Rare diseases, also related to as orphan diseases, are life-threatening or chronically debilitating conditions of different origin. Disease is considered as rare if it affects not more than 5 in 10 000 people in the EU.	- Orphan drugs (ODs) are medicinal products intended for diagnosis, prevention, or treatment of life-threatening or very serious diseases affecting less than 5 in 10 000 people in the European Union (EU).<br>- These drugs are called ,orphans, because the pharmaceutical industry has little interest, under normal market conditions, in developing and marketing products intended for only a small number of patients suffering from very rare conditions		
2014[51]	National Institute for Health and Care Excellence (NICE)	Abstract	Ultra-orphan diseases affect a very small patient population, defined by the National Institute for Health and Care Excellence (NICE) as those diseases with a prevalence of ≤ 1: 50,000. Medicines for these indications are difficult to develop in part due to challenges associated with recruiting for clinical trials from a small patient population. Within this context, global payer bodies have assessed these therapies with modified evidence requirements and opportunity for very high prices. We performed a health technology assessment (HTA) review of two ultra-orphan products – eculizumab/Soliris and iduronate-2-sulfatase (IDS)/Elaprase – to gain insight into the evolving HTA evidence requirements for ultra-orphan medicines and comparatively evaluate key decision drivers across geographies.			Ultra-orphan diseases affect a very small patient population, defined by the National Institute for Health and Care Excellence (NICE) as those diseases with a prevalence of ≤ 1: 50,000.	
2014[52]	Belgium	Qualitative research	The aim of this study is to use a combination of qualitative research methods to examine which official and non-official factors influence reimbursement decisions for orphan drugs in Belgium.	In Europe, rare diseases are defined as life-threatening or chronically debilitating diseases with a prevalence of 50 out of 100000 individuals or less.			

Year	Country/ Jurisdiction / Organization	Study design	Aim	Definition			
				RD	OD	URD	UOD
2014[53]	India, US, Europe, and Japan	Review	An attempt has been made to put forward the challenges faced by rare disease drug development and the current scenario of orphan drug legislations in India. The objective of this review is to look into Indian orphan drug regulations and an emphasis has been laid on Orphan Drugs Act (ODA) of US and orphan drug policies of other developed countries such as Europe, Japan, and Australia, thus showing the requirement of adopting ODA like legislation in India.	- In United States (US), the Orphan Drugs Act (ODA) is a federal law concerning rare diseases that affect fewer than 200,000 people or are of low prevalence (<7.5/10,000 in the community)<br>- A disease or disorder that affects fewer than 5 in 10,000 citizens is the definition for rare in Europe (Orphan Drug Regulation 141/2000)<br>- Any disease with fewer than 50,000 prevalent cases (0.4%) is Japan, definition of rare disease."			
2014[54]	USA, EU, Japan, Australia, Taiwan, South Korea, Alberta, and Ontario	Perspective-workshop	The present paper sets out to explain the rationale underlying a recent expert consensus, recommending a more rigorous assessment of the clinical effectiveness of ultra-rare disorders (URDs,) applying established standards of evidence-based medicine.	- Definitions for, orphan disorders, typically include a criterion of prevalence or incidence and differ somewhat between jurisdictions.<br>- In the USA, these are disorders with a prevalence of less than 200,000 affected persons (according to the Orphan Drug Act of 1983, and Orphan Drug Regulation of 1993)<br>- In the EU, prevalence must be less than 1 per 2000 (or less than 0.05%) of the population (according to EU Regulation CE No. 141/2000 of 2000)<br>- Strict criteria have also been set in Japan (fewer than 4 per 10,000, according to Orphan Drug Regulation of 1993)<br>- Australia (less than 1.1 per 10,000, according to Orphan Drug Policy of 1997)<br>- In Taiwan and South Korea, prevalence thresholds have been set at less than 1 per 10,000 and 1 per 20,000, respectively		- URD: conditions with a prevalence of less than 1 per 50,000 persons (NICE, Alberta). The qualifier required by AGNSS was less than 500 persons affected in England (i.e., ~1 in 100,000 of the English population). An incidence rate of fewer than 1 in 150,000 live births or new diagnoses per year in Ontario<br>- No official definition of ‚ultra-orphan disorders‚ has yet been adopted globally. Rather, this informal subcategory was introduced by the National Institute for Health and Care Excellence (formerly, the Institute for Health and Clinical Excellence, and the Institute for Clinical Excellence; NICE) conditions with a prevalence of less than 1 per 50,000 persons"	National Institute for Health and Care Excellence (formerly, the Institute for Health and Clinical Excellence, and the Institute for Clinical Excellence; NICE), drugs with indications for conditions with a prevalence of less than 1 per 50,000 persons"
2014[55]	United States	Position Statement	This article examines the trends in public discussion of high-cost drugs and the potential consequences for orphan drug development.	Prevalence of under 200,000 people in the United States	Drugs to treat conditions defined as rare, that is with a prevalence of under 200,000 people in the United States		
2015[56]	United States	Abstract	We assessed trends in approvals of new drugs with orphan indications in the US and in the prevalence of orphan drugs approved by the FDA from 1983 to 2014 compared to non-orphan drug approvals in the same time frame		Orphan drugs are indicated for rare diseases and conditions.		Indications approved for use in diseases with a prevalence of less than 1000 patients (i.e.: ultra-orphan drugs)
2015[57]	Egypt, U.S.	Chapter	We introduce in this study a system that classifies the orphan drugs according to their probability of structural similarity		- Orphan drugs are a treatment for rare diseases.<br>- Orphan drug legislation by the U.S. Food and Drug Administration (FDA) is motivating drug companies to develop drugs that have low development cost in order to treat rare diseases."		
2015[58]	United States (US) and European Union (EU),	Poster/Abstract only	The objective of this research is to identify the number of medicines that have been granted orphan designation in the United States (US) and European Union (EU) and analyse the approval trends over a ten-year time horizon with a specific focus on the number of ODs with an oncology indication.		- OD may be defined as a pharmaceutical product aimed at treating rare diseases or disorders.<br>- OD tend to consider the prevalence of the disease and the estimation of the population affected by the disease.<br>- In the USA a rare disease is defined as: <200,000 patients (<6.37 in 10,000, based on US population of 314m)<br>- In Europe a rare disease is defined as: <5 in 10,000 (<250,000 patients, based on EU population of 506m)."		
2016[59]	EU, Germany	Forum	Here we examine the factors that account for these failures and describe a variety of possible remedies. This analysis focuses on the EU perspective, though many findings are relevant toother global markets.		An orphan designation is granted to a product when the prevalence of the treated condition in the EU is not more than 5 in 10,000 or it is unlikely that marketing of the product would generate sufficient returns to justify the investment needed for its development.		
2016[60]	Italy	Review		Rare diseases (RDs), including those of genetic origin, are defined by the European Union (EU) as life-threatening or chronically			

Year	Country/ Jurisdiction / Organization	Study design	Aim	Definition			
				RD	OD	URD	UOD
				debilitating conditions whose prevalence is so low (less than 5 per 10,000)			
2016[61]	UK; (EU15 plus Nordics and Poland)	Abstract	To review HTA requirements currently in place for treatments for rare diseases in selected European countries (EU15 plus Nordics and Poland), to identify and evaluate differences between country requirements.	Definitions of orphan (prevalence ≤5:10,000)			Ultra-orphan drug (prevalence ≤1:50,000)
2016[62]	France	Poster/Abstract only	This study aims to analyse their impact on reassessment with a specific focus on orphan medicines.		Orphan designation is a status assigned to a drug intended to treat a rare condition.		
2016[63]	Japan and Europe	Model	This study focused on the difference of rare disease prevalence between Japan and Europe, classified the rare diseases comprehensively using cluster analysis and analysed the influence of prevalence on research activity and drug development.	Intractable diseases, is a Japan-specific conception of diseases with (i) unknown etiology (ii) no effective treatment, (iii) rare status (iv) necessity of long-term treatment	Designated intractable diseases over 50,000 patients were targeted for orphan drug designation in April ,2015, but were excluded due to the short implementation period). The prevalence was calculated as the rate per 100,000 population using the number of patients with the relevant disease provided by the MHLW website		
2016[64]	Asia-Pacific, Australia, Japan, Singapore, South Korea, and Taiwan	Poster/Abstract only	To evaluate the impact of national orphan drug policy and existing reimbursement mechanisms over the implementation of managed entry agreements (MEAs) for orphan drugs in the context of five Asia-Pacific countries.		- Australia: Prevalence threshold for orphan disease designation: 0.9 in 10,000<br>- Japan: Prevalence threshold for orphan disease designation: <3.9 in 10,000<br>- Singapore: Prevalence threshold: 37.7 in 10,000<br>- South Korea: Prevalence threshold: <4.0 in 10,000<br>- Taiwan: Prevalence threshold for orphan disease designation: <1 in 10,000"		
2017[65]	Spain	Abstract	Identify if the official criteria of Spanish P&R process are related with P&R approval for ODs.			Ultra-orphan diseases affecting <1/50000 inhabitants	
2017[66]	China	Commentary	The current authors proffered 300,000 to 500,000 cases as a reference threshold with which to define rare diseases in China. This proposal linked the concept of rare diseases with orphan drugs, so it is highly useful in terms of Chinese policymaking on rare diseases	- Disorders with a prevalence less than 1/500,000 or with an incidence less than 1/10,000 among new-borns<br>- More recent - 300,000 to 500,000 cases as a reference threshold with which to define rare diseases in China			
2017[67]	Bulgarian	Text and opinion	- To highlight the possible trends in the further development of requirements for orphan medicines entering the Bulgarian market on the basis of the global situation and trends."<br>- The goals of the current study are to determine the access of orphan medicines to the Bulgarian pharmaceutical market considering the currently available legislation on Health Technology Assessment (HTA) and reimbursement strategies for orphan medicines, the current number of orphan medicines included in the PDL and their total financial burden"		Orphan medicinal products (OMPs) are used for severe, life-threatening diseases with no or limited available therapeutic options		
2017[68]	Sweden	Editorial Commentary	Processes related to drug pricing, reimbursement, and thereby availability, vary between countries, thus having implications on patient care. These processes are discussed, with specific focus on three drugs used in paediatric nephrology: a galsidase beta (for Fabry disease), eculizumab (for atypical haemolytic uremic syndrome), and cysteamine bitartrate (for cystinosis).	Rare diseases are severe, chronic, debilitating, and/or life-threatening conditions that are often hereditary and, by definition, affect less than 1 in 2000 individuals in the European Union, or fewer than 200,000 individuals in the USA, at any given time		Ultra-rare diseases have a prevalence of 1 in 50,000 individuals or less in Europe (EU regulation 536/2014).	
2017[69]	French	Poster/Abstract only	- To explore French stakeholders, policy, implicit or explicit, toward orphan drugs on both Transparency Committee (TC) assessment and pricing decisions<br>- To compare authorities, decisions between two periods of time (2006-2010 and 2011-2016) in order to describe variations on assessment and price lifecycle."	In Europe orphan disease is defined by a prevalence of less than 5 in 10 000 inhabitants which represent a maximum target population of 30 000 patients in France.	An orphan drug is a pharmaceutical agent that has been developed specifically to treat a rare disease, itself referred to as an orphan disease. Often severe and disabling, affecting a limited number of people (the threshold admitted for the prevalence is 1 in 2000 in Europe).		
2017[70]	Europe	Book - Chapter	Is to bring together the necessary elements for an efficient overall strategy, hence the adoption of Commission Communication COMM (2008) 679 final on 11 November 2008<br>1. Making rare diseases more visible<br>2. Encouraging Member States to develop national rare diseases plans in their health policies.<br>3. Providing European support and cooperation, such as ensuring that common policy guidelines are developed and shared	Rare diseases, are defined by the European Union as life-threatening or chronically debilitating diseases with low prevalence (less than 5 per 10,000).			

Year	Country/ Jurisdiction / Organization	Study design	Aim	Definition			
				RD	OD	URD	UOD
2017[71]	UK, England, and Wales	Poster/Abstract only	The objective of this study was to evaluate National Institute for Health and Care Excellence Highly Specialised Technology (NICE HST) programme evaluations in the context of the changes and assess the potential impact they may have on patient access to ultra-orphan treatments in England and Wales			Ultra-orphan conditions are defined as diseases affecting <1000 people in England and Wales by the National Institute for Health and Care Excellence (NICE)	
2017[72]	Europe	Research article	Our multidisciplinary working group discussed the most relevant clinical and economic issues that are perceived to complicate the cost-effectiveness evaluation of orphan diseases and orphan medicinal products and to drive the high ICERs. Subsequently potential policy approaches are presented.	Orphan disease is defined in the EU Orphan Regulation 141/2000 (10) as:<br>1. A disease that is Life-threatening or chronically debilitating.<br>2. Prevalence of the condition in the EU of less than 5 in 10,000 or unlikely that marketing of the medicine would generate sufficient returns to justify the investment needed for its development; and<br>3. No satisfactory method of diagnosis, prevention or treatment of the condition concerned has been authorized in the EU, or, if such a method exists, the medicine must be of significant benefit to those affected by the condition.			
2017[73]	UK	Research	The aims of this study were to apply the MCDA framework that was proposed by Hughes-Wilson et al. (Orphanet J Rare Dis 7:74, 2012) to a range of orphan drugs in different diseases, with a view to testing the relationship between drug price and aggregated MCDA scores for each product.	Disease with a prevalence of 1 per 2,000 or less			
2018[74]	Sweden	Review	In this work we provide an up-to date analysis of drug target interactions for approved and clinical trial drugs and examine the major developments and trends in pharmaceutical development	Rare diseases are defined in the US as a disease or condition affecting less than one in 200 000 people.	Orphan drugs encompass pharmaceuticals that are intended to treat these types of diseases		
2018[75]	Poland, Netherlands, and Russia	Review	The goal of this article is to provide an in-depth review of rare disease policies and the reimbursement of ODs in 3 European countries, two EU members (Poland, the Netherlands) and a non-EU one (Russia).	Poland uses the EU definition of rare disorders, which considers a disease as rare if it affects less than 1 in 2000 people (< 5 in 10000 people)		Ultra-rare being <1 in 50000 people'	
2018[76]	Poland	Systematic review	The goal of this article is to provide an overview of the current state of knowledge and latest developments in the field of MCDA in HTA for orphan drugs, to review existing models, their design characteristics, as well as to identify opportunities for further model improvement.		The disease prevalence threshold in the EU for an orphan drug designation is well-defined at ≤ 5 per 10.000.		
2018[77]	China	Research	The primary objectives are to establish standardization for registration platform, to build biobanks of genomic data, and to create partnerships for data sharing and research collaboration	The United States defines rare diseases as disorders affecting fewer than 200,000 individuals while the European definition is diseases with a prevalence of lower than 5/10,000.	In 2010, at a seminar conducted by the Genetics Society of the Chinese Medical Association, experts mainly in the field of medical genetics proposed that (any diseases of prevalence lower than 1/500,000 in the overall population or 1/10,000 among new-born's should be considered as a rare disease).		
2018[78]	UK, Scotland	Review	This review identified special HTA, and reimbursement considerations introduced for assessment of orphan drugs and implications for manufacturers.		- According to the European Medicines Agency definition, orphan drugs are intended for diagnosis, prevention, or treatment of rare diseases whose conditions affect no more than 5 in 10,000 persons.<br>- OD proven at marketing authorization if the annual budget impact is less than €30 million per year for a particular indication.<br>- Certain special HTA criteria are applied to orphan drugs:<br>1. Higher P values for small sample sizes<br>2. Use of surrogate endpoints<br>3. Additional benefit is considered proven if the budget impact is less than €50 million per year for a particular indication.<br>- Higher therapeutic benefit is automatically recognized for orphan drugs because these drugs had to prove significant additional therapeutic benefit compared with other possibly already approved drugs as part of the European marketing authorization procedure.		- Currently, no official definition of "ultra-orphan disorders" has been adopted globally. This informal subcategory was introduced by the National Institute for Health and Care Excellence (NICE), which applied it to drugs with indications for conditions with a prevalence of less than 1 per 50,000 persons.<br>- In October 2018, a process will be introduced to allow faster access to ultra-orphan drugs: •The Scottish government will introduce a new definition of ultra-orphan medicines that can treat very

Year	Country/ Jurisdiction / Organization	Study design	Aim	Definition			
				RD	OD	URD	UOD
							rare conditions affecting fewer than 1 in 50,000 people—approximately 100 people or fewer in Scotland
2018[79]	Taiwan, United States, EU, and Japan	Research article	- The objectives of this study were to examine 2003,2014 longitudinal trends in the prevalence and expenditure of rare diseases in Taiwan. We also analysed these trends for two specific rare diseases, amyotrophic lateral sclerosis (ALS) and multiple sclerosis (MS), because ALS is the main targeted rare disease in the ice bucket challenge activity, and MS is another rare disease with similar symptoms to those of ALS.<br>- This study examined the national trends in the prevalence of rare diseases and their health-related economic burden (including medication costs) in Taiwan.	- The general definition of a rare disease in Taiwan is <1/10,000 persons.<br>- In the United States and Japan, a rare disease is one with a prevalence of fewer than 200,000 persons and 50,000, respectively. The EU defines rare diseases as fewer than 5 per 10,000 persons			
2018[80]	UK, England	Poster/Abstract only	This research aims to identify, compare, and evaluate willingness to pay (WTP) thresholds across countries		WHO recommends a WTP of <3 times GDP per capita/QALY		HST for ultra-orphan indications Euro113,900-341,700/QALY in England
2018[81]	Germany	Review	- The valid guidelines and the regulations of the German health system are discussed in this article.<br>- The criteria for indication and monitoring of off-label use are shown, especially focused on the problem of refractory myasthenia gravis.	- Since 2000, diseases with a prevalence of < 5 out of every 10,000 people in the EU have been defined as "rare diseases."<br>- According to a statement by Orphanet regarding **myasthenia gravis** in Europe, this amounts to a prevalence of 1–9/100,000 population.		Rare diseases are "singular cases" or "individual cases", which are considered "ultra-rare diseases" (prevalence: <1:10,000), including, for example MuSK-positive myasthenia gravis (prevalence 0.05–0.65/100,000 or congenital myasthenic syndrome (CMS)	
2018[82]	United States	Abstract	To estimate the pharmacy budget impact (per member per month [PMPM]) of five orphan drugs with single chronic indications.	There are up to 7,000 rare diseases. defined as a condition affecting fewer than 200,000 people.			
2018[83]	Canada, Scotland, Australia, and New Zealand	Research	The objective of the present study was to analyse the basis for Common Drug Review (CDR) orphan drug recommendations and to compare recommendations to those in other jurisdictions. In the current study we have reviewed CDR recommendations for orphan drugs, defined the parameters involved in decision making, and compared recommendations with those made in Scotland, Australia, and New Zealand.	- (Canada) proposed definition of a rare or orphan disease as one that affects < 1in 2000 persons, a definition aligned to that used in the European Union<br>- Approximately 7000 such diseases have been identified and it is estimated that 1 in 12 Canadians, or about 2.8 million individuals, may be living with a rare disease			
2018[84]	Spain	Meeting Abstract	This presentation will review these forces and the multiple business models for pursuing orphan indications that they offer and discuss some of the unique scientific and business aspects that make the orphan space unique, including the crucial central role of rare disease patient organizations.	Rare diseases, which are those affecting <5 in 10,000 people in Europe.			
2018[85]	France	Poster/Abstract only	The aim of this analysis was to discuss ICERs of orphan drugs and their characterizations issued by the CEESP		Orphan drugs according to the Transparency Committee opinions and designations are typically indicated in conditions that have a prevalence of below 5 in 10,000		
2018[86]	Japan	Symposium	Overview the designation and supporting systems for development of orphan drugs in Japan and foreign country, and introduce our experience of promoting the orphan drug in neuromuscular fields	- Rare diseases are any diseases that affected the relatively small number of patients, and generally chronically debilitating, life threatening.<br>- Rare disease is definitely in the space of unmet medical needs.	Orphan drugs, which are the drugs for rare diseases		
2018[87]	United States	Review	The purpose of this study was to compare published ICER estimates, as a measure of relative value, across several orphan drugs which are indicated to treat rare diseases in paediatrics and adults.	A rare disease was defined as a condition with a prevalence of ≤620/million persons.		Ultra-rare diseases (affecting <20/million persons)"	
2019[88]	United States, WHO, and Europe	Book - chapter		- WHO, orphan disease refers to a disease with a low prevalence of less than 6.5–10 cases in 10,000 people.<br>- USA, orphan disease is defined as one that affects less than 200,000 individuals.<br>- Europe, disease with prevalence of less than 5 in 10,000 people	- Orphan drugs are defined as the drugs used for the diagnosis, prevention, or treatment of orphan disease.<br>- Orphan drugs are those drugs having both orphan and non-orphan indications		
2019[89]	UK	Model	- Our study tested the criteria preferences and possibilities for implementation of the EVIDEM MCDA framework for orphan drugs with a diverse group of 140 stakeholders in Kazakhstan,	Diseases that are life-threatening or chronically debilitating are qualified as rare diseases (RD) in the EU if their prevalence is <5 per 10.000			

Year	Country/ Jurisdiction / Organization	Study design	Aim	Definition			
				RD	OD	URD	UOD
			Netherlands, Poland, Romania, Russia, Turkey, and Ukraine (KZ, NL, PL, RO, RU, TR, UA).<br>- The purpose of the study was to perform a weight elicitation for the EVIDEM rare disease model (v3.0) in a wider region in Eurasia with a sizeable group of experts (100-200), in order to identify key differences between countries and types of stakeholders as well as to compare weighting results from other studies. A secondary goal was to test the usefulness of a questionnaire tool designed for this purpose.				
2019[90]	UK	Abstract			- For a drug to be appraised via the HST process it must meet seven criteria, based on: a small and clinically distinct patient population, a limited number of specialist treatment centres for the indication in question, treatment price, and severity of the condition.<br>- The current NICE appraisal system means orphan drugs that do not meet HST criteria go through the standard technology appraisal (TA) process, with a cost-effectiveness threshold of¬£30 k/QALY, or¬£50 k/QALY when end-of-life criteria are met		
2019[91]	UK	Poster/Abstract only	This research compares NICE Highly Specialised Technologies (HST) appraisal outcomes with corresponding guidance by other European HTA bodies, stratified by payer archetype: cost-effectiveness versus clinical effectiveness			Ultra-orphan disease (prevalence: <1:50,000)	
2019[92]	Italy	Meeting Abstracts	This paper aims to give some insights into the Italian Pricing & Reimbursement (P&R) Policies on Orphan Medical Products (OMPs) highlighting the strengths and weaknesses of the system.		- OMPs are drugs intended for the treatment of serious conditions affecting less than 5 in 10,000 people in the EU.<br>- AIFA may grant a medicine the status of innovative drug according to 3 criteria: unmet medical needs, clinical added value, and quality of evidence.		
2019[93]	UK (England and Scotland)	Review/ Poster	This research reviewed recent assessments of orphan and ultra-orphan drugs by NICE and the SMC, and disparities in availability for NHS patients between England and Scotland.		Treatments for diseases with a prevalence of <5 in 10,000 in the EU, which are life-threatening or severely disabling and have no satisfactory treatment available, are granted orphan designation by the European Medicines Agency (EMA)		The NICE Highly Specialised Technology Programme (HSTP) and the SMC consider ultra-orphan to be <1 in 50,000 and meeting other specialised criteria. "
2019[94]	UK	Review	This review provides an overview of NIBSC, work in rare diseases and highlights the positive impact of the work of standardization institutions in this field	Rare diseases are defined as conditions not affecting more than 5 in 10,000 people in Europe			
2019[95]	Spain	Review	The present study aims to develop a reflective MCDA framework, based on EVIDEM methodology, with relevant criteria that allows the evaluation and positioning of OD to aid decision-making at the national level in Spain.		Orphan Drugs (ODs) are intended for the diagnosis, prevention, or treatment of life-threatening or very serious conditions that affect no more than 5 in 10,000 (rare diseases) in the European Union (EU).		
2020[96]	India, Organization for Rare Diseases India (ORDI), WHO, EU, US, Japan, and Australia	Review	This review provides a brief account on RDs and their prevalence, followed by a discussion on the major RDs-associated challenges in general, an account on the methods that can be adopted for conducting fruitful molecular genetic studies of monogenic diseases, and the experiences of genetic research in Indian context with a special reference to a genetically vulnerable and low resource region like J&K - India.	- Organization for Rare Diseases India (ORDI) has suggested a threshold for defining a disease as rare if it afflicts 1 in 5,000 individuals in India.<br>- The base prevalence rate of RDs set by the World Health Organization (WHO) is approximately 1 in 2,000 people.<br>- A genetic disorder prevalent in the European Union (EU) is considered rare only if it affects 5 or less per 10,000 cases, whereas the incidence rate for RDs in the United States is 7 or less per 10,000 individuals. These numbers translate to nearly 30 million Europeans and 25 million North Americans (approximately 1 in every 10) affected by any of the known RDs.<br>- The incidence rate is estimated to be ≤2.5 cases in 10,000 and 1 in 10,000 individuals for Japan and Australia, respectively			
2020[97]	Belgium	Position Statement	The current paper aims to set a further step and translate the findings and recommendations from the many existing initiatives into a pragmatic and realistic methodology. The proposed tool will provide guidance to inform multi-stakeholder discussions and		Many of the treatments developed for rare diseases will have an Orphan Medicinal Product (OMP) designation indicating that they are likely to deliver benefit in an area		

Year	Country/ Jurisdiction / Organization	Study design	Aim	Definition			
				RD	OD	URD	UOD
			reimbursement decision making about specialised treatments for rare diseases." "Additionally, the paper provides guidance on the potential of Real-World Evidence (RWE) ,i.e., data collected outside the context of RCTs to help address such uncertainties.		of high unmet need. Their approval may be based on a small or uncontrolled trial		
2020[98]	Western Eurasian region: Armenia, France, Germany, Kazakhstan, Latvia, The Netherlands, Poland, Romania, Russia, Turkey, Ukraine, and the United Kingdom.	Systematic Review	This study aimed to create a comprehensive and in-depth overview of rare diseases policies and reimbursement of OMPs in a selection of 12 countries in the Western Eurasian region: Armenia, France, Germany, Kazakhstan, Latvia, The Netherlands, Poland, Romania, Russia, Turkey, Ukraine, and the United Kingdom. the aim of this article is to bridge the identified gaps by presenting an overview and comparison of current rare disease policies, HTA and reimbursement processes for orphan drugs in a broader range of Eurasian countries.	- The EU has officially defined rare diseases as being rare when they affect fewer than 1 in 2000 (i.e., a prevalence of 5 or less per 10,000) and in most of the selected countries this definition is used [FR, DE, LV, NL, PL, RO, UK, and UA<br>- In Russia the maximum prevalence for a rare disease is defined as 1 in 10,000<br>- Some countries use additional definitions in situations where a condition is not officially defined as rare, such as in the UK, where the National Health Service (NHS) classifies all conditions that require specialized medical care also as rare if they occur in <500 citizens yearly.<br>- Turkey defines a rare disease when they affect no more than 1 in 100,000, which is 50 times less frequent than the European Union definition.<br>- There is no specific definition for ‚rare disease‚ in Armenian legislation, only ‚levels of disability, which define whether the patient will receive the necessary medicines for free or not	The Netherlands defines the classification ‚orphan drug‚ as either having an official EU orphan designation or if it targets a disease with a prevalence of <1 in 150,000 and shows a clinically proven therapeutic benefit and no other registered medicine exists.		Effective from October 2018, Scotland has introduced a new definition for ultra-orphan drugs: ‚medicines that are used to treat a condition with a prevalence of 1 in 50,000 or less or around 100 people in Scotland, which will mostly be used to facilitate early access programs and reimbursement processes
2020[99]	France	Review	To detect among the drugs approved for limited populations any impact of the orphan status on the assessment outcome of medical benefit (SMR) or improvement in medical benefit (ASMR) carried out by the French authority for health (HAS)	Prevalence of rare disease < 5/10 000 as per EMA"	An orphan designation is granted by EMA for all new drug intended to treat a life-threatening or chronically debilitating disease, provided a maximum prevalence in Europe of 5/10,000 and when no satisfactory alternative method can be authorised, or, if such a method exists, the medicine must be of significant benefit to patients.		
2020[100]	UK	Commentary	This paper explores the successes and limitation of both the regulation and its implementation mechanisms in the current regulatory context, and suggests some improvements that could maximise its benefits and boost rare disease research even further	- Rare diseases are categorized as ‚orphan diseases‚ because their occurrence in a small number of patients means that, despite apparent high unmet medical need, there is limited scientific understanding, making it difficult to justify the development risk and investment to develop new treatments.<br>- The European Union defines a rare (or ‚orphan‚) disease as a life-threatening or chronically debilitating disorder that affects <5 in 10,000 people in the European Union.		Prevalence can be much lower, leading to the concept of the‚ ultra-orphan disease‚ for diseases with an estimated prevalence of <1 in 50,000 people	
2020[101]	India	Abstract	The purpose of this paper is to identify the hurdles in the field of orphan drugs in India and suggest solutions to address the same.	An orphan disease is defined as a condition that affects fewer than200,000 people nationwide	Orphan Drug is used to treat such a condition.		
2020[102]	India	Review	To understand orphan drugs and national policy on treatments of rare diseases. To overview the condition for pricing of orphan drugs in India and government schemes which are helping out for patient needs. To highlight the need of regulations on orphan drugs for sale and manufacture of orphan drugs in India.	A rare disease is a health disorder of low occurrence that affects a limited number of people in the general population as opposed to other prevalent diseases.	Orphan drugs are the drugs and natural products used in treatment, diagnosis, or prevention of rare disease.		
2020[103]	194 World Health Organization member countries and other areas (Hong Kong, Kosovo, Macau, Palestine, Sahrawi, Republic, Philippines and Taiwan)"	Health Policy Analysis	This study aims to provide an up-to-date global overview of ODP (Orphan drug policies) in the era of innovative medicine and to reflect associated changes in drug regulation policy. This review provides an overview of global policies that optimize development, licensing, pricing, and reimbursement of orphan drugs.	- Rare diseases are typically defined as conditions with limited treatment alternatives, with an average prevalence of fewer than 40 to 50 cases per 100 000 population or that affect a small number of patients compared with the total population.<br>- When defining rare diseases, most countries/ areas adhered to the European Union definition of low prevalence (0.05%), whereas others followed the number of prevalent cases, such as Australia (< 2000), South Korea (<20 000), and the United States (<200 000). Countries/areas such as Chile, Kenya, Peru, and Singapore required the disease severity to be, life threatening, and severely- or chronically-‚debilitating.<br>- Rare disease or condition, means any disease or condition which (A) affects less than 200,000 persons in the United States, or (B) affects more than 200,000 in the United States and for which there is no reasonable expectation that the cost of developing and making available in the United States a drug for such disease or	- Orphan drugs are often defined as drugs intended for the treatment, diagnosis, prophylaxis, or rehabilitation of rare diseases.<br>- Orphan drugs are also defined by their availability as pharmaceutical products or active ingredients not developed, imported, or registered owing to low commercial returns and unfavorable marketing conditions. Countries/areas such as China and Vietnam acknowledged orphan drug designation from referenced competent authorities. A medicinal product shall be designated as an orphan medicinal product if its sponsor can establish:<br>(a) that it is intended for the diagnosis, prevention or treatment of a life-threatening or chronically debilitating condition affecting not more than five in 10 thousand persons in the community when the application is made, or that it is intended for the		

Year	Country/ Jurisdiction / Organization	Study design	Aim	Definition			
				RD	OD	URD	UOD
				condition will be recovered from sales in the United States of such drug (United States)<br>- Designation of rare diseases: The DOH, upon recommendation of the RDTWG, shall have the authority to designate any disease that is recognized to rarely afflict the population of the country as a rare disease. (The Philippines)	diagnosis, prevention or treatment of a life-threatening, seriously debilitating or serious and chronic condition in the community and that without incentives it is unlikely that the marketing of the medicinal product in the community would generate sufficient return to justify the necessary investment; and<br>(b) that there exists no satisfactory method of diagnosis, prevention, or treatment of the condition in question that has been authorized in the Community or, if such method exists, that the medicinal product will be of significant benefit to those affected by that condition<br>- In order to obtain the designation of a medicinal product as an orphan medicinal product, the sponsor shall submit an application to the Agency at any stage of the development of the medicinal product before the application for marketing authorization is made. European Union		
2020[104]	Santiago de Chile	Book - Chapter		- Rare diseases (RDs) or orphan diseases, by definition, are conditions that affect a small number of individuals most RDs are chronic and debilitating and are a substantial cause for disability and early death.<br>- Based on Orphanet, disease inventory, it is evident that the majority of RDs are of genetic etiology, and a smaller percentage is autoimmune or infectious disorders, in addition to some rare cancers."<br>- RDs are a highly heterogeneous group of disorder			
2020[105]	China, Australia, Japan, South Korea, and Taiwan	Poster/Abstract only	We sought to identify the regulations and policies related to market access for orphan drugs in five major markets from the APAC Region, with the aim of providing an overview of the factors designed to support sponsors of orphan medicinal products. Specifically, we focused on policies in Australia, China, Japan, South Korea, and Taiwan	- "China: Rare disease defined as that affecting less than 1 per 500,000 population.<br>- South Korea: Rare disease defined as that affecting: Less than 20,000 people in Korea (i.e., <4 per 10,000 population)<br>- Japan: Rare disease defined as that affecting: Less than 50,000 people in Japan (i.e., <4 per 10,000 population).<br>- Taiwan: Rare disease defined as that affecting less than 1 per 10,000 population.<br>- Australia: Rare disease defined as that affecting less than 5 per 10,000 population"			
2021[106]	South Korea	Expert Opinion	This paper reviews key factors that should be considered in the process of development, regulation, and market access of orphan drugs in South Korea with a particular focus on the pricing and reimbursement review process.		In South Korea, the Korea Ministry of Food and Drug Safety formulates ODs, which should satisfy two conditions related to the number of patients and the existence of alternatives. In other words, drugs used for a disease with 20,000 or fewer patients (population with the disease) and diseases for which adequate treatments or drugs have not yet been developed, or drugs that significantly improve safety or efficacy compared to existing alternatives, are designated as OD.		
2021[107]	UK	Review	This review provides an overview of the strengths and limitations of value assessment frameworks (VAFs) for the reimbursement of orphan drugs in Europe and may serve as a guide for decision-makers.	- Rare diseases are a group of diverse diseases, each characterized with low prevalence: occurring in less than one in 2,000 people in Europe.<br>- They are defined as life-threatening or chronically debilitating, and are mostly caused by a genetic predisposition	The, Orphan Medicinal Product Regulation, defines OMPs as products for the ,diagnosis, prevention, or treatment of life-threatening or very serious conditions that affect no more than 5 in 10,000 people in the European Union		
2021[108]	Spain	Research	This study aimed to determine the most relevant criteria for the reimbursement of OMPs in Spain, from a multi-stakeholder perspective, and using multi-criteria decision analysis (MCDA). The objective of this study was twofold: first, to review, discuss, and reach a consensus on the most relevant criteria for decision-making about pricing and financing OMPs in Spain; and second, to prioritize them according to their relative importance based on	- Rare diseases are diseases of low prevalence and high complexity that can lead to death or chronic disability.<br>- In Europe, rare diseases are defended as those pathologies that affect less than 5 people per 10,000 inhabitants.	Orphan medicinal products (OMPs), which are intended to diagnose, prevent, or treat rare diseases, have a shared community procedure for being designated as such in the European Union, and this community approach provides opportunities for research, development, and marketing	Ultra-rare, affecting less than 1 person per 50,000 inhabitants."	

Year	Country/ Jurisdiction / Organization	Study design	Aim	Definition			
				RD	OD	URD	UOD
			the preferences stated by different stakeholders, following the MCDA methodology.				
2021[109]	New Zealand	Online survey	The objectives of this study were to measure the relative societal importance of values of New Zealanders in informing drug funding decisions and to determine how New Zealanders trade of funding in various scenarios between common and rare diseases.	A rare disorder is defined by PHARMAC (the Pharmaceutical Management Agency) as affecting less than 1:50,000 people in the New Zealand population, which is a considerably lower prevalence threshold than other nations that are from 5 to 76 per 100,000 people			
