# Supplementary material for: Global insight into rare disease and orphan drug definitions: a systematic literature review
Source: BMJ Open. 2025 Jan 25;15(1):e086527. doi: 10.1136/bmjopen-2024-086527 (PMC11784410; doi:10.1136/bmjopen-2024-086527)
Supplement: online supplemental file 4 [file bmjopen-15-1-s004.pdf]

Supplementary Table 4: Critical Appraisal Result

Critical Appraisal Result for Systemic Reviews and Research Syntheses studies

Studies	Q1) Is the review question clearly and explicitly stated?	Q2) Were the inclusion criteria appropriate for the review question?	Q3) Was the search strategy appropriate?	Q4) Were the sources and resources used to search for studies adequate?	Q5) Were the criteria for appraising studies appropriate?	Q6) Was critical appraisal conducted by two or more reviewers independently?	Q7) Were there methods to minimize errors in data extraction?	Q8) Were the methods used to combine studies appropriate?	Q9) Was the likelihood of publication bias assessed?	Q10) Were recommendations for policy and/or practice supported by the reported data?	Q11) Were the specific directives for new research appropriate?
1. 2018 [60]	Yes	Yes	Yes	Yes	Yes	Yes	Yes	Yes	Yes	Yes	Yes
2. 2020 [84]	Yes	Yes	Yes	Yes	Yes	Yes	Yes	Yes	Yes	Yes	Yes
3. 2021 [110]	Yes	Yes	Yes	Yes	Yes	Yes	Yes	Yes	Yes	Yes	Yes

1. Critical Appraisal Result for Text Opinion studies

Studies	Q1) Is the source of the opinion clearly identified?	Q2) Does the source of opinion have standing in the field of expertise?	Q3) Are the interests of the relevant population the central focus of the opinion?	Q4) Is the stated position the result of an analytical process, and is there logic in the opinion expressed?	Q5) Is there reference to the extant literature?	Q6) Is any incongruence with the literature/sources logically defended?
1.2003 [3]	Yes	Yes	Yes	Yes	Yes	Yes
2.2005 [5]	Yes	Yes	Not applicable	No	Yes	Yes
3.2006 [7]	Yes	Yes	Yes	Not applicable	Yes	No
4.2009 [9]	Yes	Yes	Yes	Not applicable	Yes	Not applicable
5.2010 [11]	Yes	Yes	Yes	Yes	Yes	No
6.2010 [12]	Yes	Yes	Unclear	No	Yes	No
7.2014 [33]	Yes	Yes	Yes	Yes	Yes	Yes
8.2017 [51]	Yes	Yes	Yes	Yes	Yes	Yes
9.2017 [111]	Yes	Yes	Yes	Yes	Unclear	NO
10. 2019 [78]	Yes	Yes	Yes	NO	Yes	Yes
11. 1992 [1]	Yes	No	Yes	NO	Yes	Not applicable
12. 2004	Yes	Yes	Yes	Yes	Yes	Not applicable
13. 2008 [8]	Yes	Yes	Yes	Yes	Yes	NO
14. 2010 [13]	Yes	Yes	NO	NO	Yes	Not applicable

15. 2011 [15]	Yes	Yes	Yes	Yes	Yes	NO
16. 2013 [25]	Yes	Yes	Yes	Yes	Yes	NO
17. 2013 [28]	Yes	Yes	Yes	Yes	Yes	NO
18. 2014 [37]	Yes	Yes	Yes	Yes	Yes	NO
19. 2016 [44]	Yes	Yes	NO	Yes	Yes	NO
20. 2018 [55]	Yes	Yes	Yes	Yes	Yes	Yes
21. 2018 [59]	Yes	Yes	Yes	Yes	Yes	NO
22. 2018 [65]	Yes	Yes	NO	Yes	Yes	NO
23. 2020 [80]	Yes	Yes	Yes	Yes	Yes	NO
24. 2020 [86]	Yes	Yes	Yes	Yes	Yes	NO
25. 2020 [112]	Yes	Yes	Yes	Yes	Yes	NO
26. 2020 [88]	Yes	Yes	Yes	Yes	Yes	NO
27. 2021 [91]	Yes	Yes	Yes	Yes	Yes	Yes
28. 2010 [14]	Yes	Yes	NO	Yes	Yes	No applicable
29. 2018 [61]	Yes	Yes	Yes	Yes	Yes	NO
30. 2021 [91]	Yes	Yes	Yes	Yes	Yes	NO

2. Critical Appraisal Result for Economic Evaluations studies

Studies	Q1) Is there a well-defined questio n?	Q2) Is there comprehensive description of alternatives?	Q3) Are all important and relevant costs and outcomes for each alternative identified?	Q4) Has clinical effectiveness been established?	Q5) Are costs and outcomes measured accurately?	Q6) Are costs and outcomes valued credibly?	Q7) Are costs and outcomes adjusted for differential timing?	Q8) Is there an incremental analysis of costs and consequences?	Q9) Were sensitivity analyses conducted to investigate uncertainty in estimates of cost or consequences?	Q10) Do study results include all issues of concern to users?	Q11) Are the results generalizabl e to the setting of interest in the review?
1.2012 [21]	Yes	Not applicable	Not applicable	Not applicable	Not applicable	Not applicable	Not applicable	Not applicable	Not applicable	Yes	Yes
2.2014 [34]	Yes	Yes	Yes	Not applicable	Yes	Yes	Yes	Yes	Not applicable	Yes	Yes
3.2014 [38]	Yes	Yes	Yes	Yes	Yes	Yes	Yes	Yes	Yes	Yes	Yes
4.2018 [63]	Yes	Yes	Yes	Yes	Yes	Yes	Yes	Yes	Yes	Yes	Yes
5.2018 [67]	Yes	Yes	Yes	Yes	Yes	Not applicable	Not applicable	Yes	No	Yes	Yes
6.2017 [57]	Yes	Yes	Yes	Yes	Yes	Unclear	NO	NO	NO	Yes	Yes

3. Critical Appraisal Result for Analytical Cross-Sectional Studies

Studies	Q1) Were the criteria for inclusion in the sample clearly defined?	Q2) Were the study subjects and the setting described in detail?	Q3) Was the exposure measured in a valid and reliable way?	Q4) Were objective, standard criteria used for measurement of the condition?	Q5) Were confounding factors identified?	Q6) Were strategies to deal with confounding factors stated?	Q7) Were the outcomes measured in a valid and reliable way?	Q8) Was appropriate statistical analysis used?

. 2012 [20]	Yes	Yes	Yes	Yes	Yes	Yes	Yes	Yes
. 2015 [41]	Yes	Not applicable	Not applicable	Not applicable	Not applicable	Not applicable	Yes	Unclear

4. Critical Appraisal Result for Qualitative Research studies

Studies	Q1) Is there congruity between the stated philosophical perspective and the research methodology?	Q2) Is there congruity between the research methodology and the research question or objectives?	Q3) Is there congruity between the research methodology and the methods used to collect data?	Q4) Is there congruity between the research methodology and the representation and analysis of data?	Q5) Is there congruity between the research methodology and the interpretation of results?	Q6) Is there a statement locating the researcher culturally or theoretically?	Q7) Is the influence of the researcher on the research, and vice- versa, addressed?	Q8) Are participants, and their voices, adequately represented?	Q9) Is the research ethical according to current criteria or, for recent studies, and is there evidence of ethical approval by an appropriate body?	Q10) Do the conclusions drawn in the research report flow from the analysis, or interpretation, of the data?
1. 2014 [36]	Yes	Yes	Yes	Yes	Yes	Yes	Yes	Yes	Yes	Yes
2. 2021 [92]	Yes	Yes	Yes	Yes	Yes	Yes	Yes	Yes	Not applicable	Yes
3. 2021 [93]	Yes	Yes	Yes	Yes	Yes	Yes	Yes	Yes	Not applicable	Yes
4. 2013 [30]	Yes	Yes	Yes	Yes	Yes	Not applicable	Not applicable	Not applicable	Not applicable	Yes
5. 2019 [59]	Yes	Yes	Yes	Yes	Yes	NO	NO	Yes	NO	Yes

5. Critical Appraisal Result for Prevalence Studies

Studies	Q1) Was the sample frame appropriate to address the target population?	Q2) Were study participants sampled in an appropriate way?	Q3) Was the sample size adequate?	Q4) Were the study subjects and the setting described in detail?	Q5) Was the data analysis conducted with sufficient coverage of the identified sample?	Q6) Were valid methods used for the identification of the condition?	Q7) Was the condition measured in a standard, reliable way for all participants?	Q8) Was there appropriate statistical analysis?	Q9) Was the response rate adequate, and if not, was the low response rate managed appropriately?
1. 2016 [47]	Yes	Yes	NO	Yes	Yes	Yes	Yes	Yes	Yes
2. 2013 [26]	Yes	Yes	Unclear	Yes	Yes	Yes	Yes	Yes	Not applicable

6. Critical Appraisal Result for Cohort Studies

Studies	Q1) Were the two groups similar and	Q2) Were the exposures measured	Q3) Was the exposure	Q4) Were confounding	Q5) Were strategies to deal with	Q6) Were the groups/participants free of the	Q7) Were the outcomes	Q8) Was the follow up time reported and	Q9) Was follow up complete, and if not, were	Q10) Were strategies to address	Q11) Was appropriate

	recruited from the same population?	similarly to assign people to both exposed and unexposed groups?	measured in a valid and reliable way?	factors identified?	confounding factors stated?	outcome at the start of the study (or at the moment of exposure)?	measured in a valid and reliable way?	sufficient to be long enough for outcomes to occur?	the reasons to loss to follow up described and explored?	incomplete follow up utilized?	statistical analysis used?
1. 2018 [61]	Not applicable	Yes	Yes	NO	NO	Yes	Unclear	NO	NO	Yes	Not applicable
