# Supplementary material for: Global insight into rare disease and orphan drug definitions: a systematic literature review
Source: BMJ Open. 2025 Jan 25;15(1):e086527. doi: 10.1136/bmjopen-2024-086527 (PMC11784410; doi:10.1136/bmjopen-2024-086527)
Supplement: online supplemental file 5 [file bmjopen-15-1-s005.pdf]

Supplementary Table 5: RDs definitions based on continents

Continent	Country, frequency	# of articles; (%)		(RD) definition	Date	Adopted / developed
North America	US (25)	24 (26%)	Orphan Drug Regulation	Defines RD according to prevalence: “rare disease’ means any disease or condition that affects less than 200000 persons in the USA’.	1993	developed
			RDA		2002	
			ODA	Defined RDs based on qualitative descriptors as follows: ‘the term ‘rare disease or condition’ means any disease or condition which occurs so infrequently in the USA that there is no reasonable expectation that the cost of developing and making available in the USA a drug for such disease or condition will be recovered from sales in the USA of such drug’.	1983	
			FDA	Define RD as ‘any disease or condition that affects less than 200000 people in the USA or affects >200000 in the USA and for which there is no reasonable expectation that the cost of developing and making available in the USA a drug for such disease or condition will be recovered from sales in the USA of such drug’		
	Canada (3)	2 (2%)	CORD	Rare disease as one that afflicts less than 1 person in 200 000.		Aligned to EU
				Estimated that 1 in 12 Canadians, or about 2.8 million individuals, may be living with a rare disease		
South America	Chile (1)	1 (1%)		Required the disease severity to be ‚life threatening, and severely- or chronically-, debilitating.		
	Peru (1)					
Europe	UK (3)	2 (2%)	the Rare Disease Framework	Defined RD based on prevalence, as a condition affecting fewer than 1 in 2000 people. (i.e., a prevalence of 5 or less per 10,000)	2021	
			NHS	Some countries use additional definitions in situations where a condition is not officially defined as rare. classifies all conditions that require specialized medical care as rare if they occur in <500 citizens yearly		
	EU (36)	35 (38%)		Rare diseases, including those of genetic origin, are life-threatening or chronically debilitating diseases which are of such low prevalence (less than 5 per 10,000persons in the European Union) that special combined efforts are needed to address them so as to prevent significant morbidity or perinatal or early mortality or a considerable reduction in an individual's quality of life or socio-economic potential.		
			European Commission on Public Health	Defines rare diseases as ‚life-threatening or chronically debilitating diseases which are of such low prevalence that special combined efforts are needed to address them.		
			Orphan Drug Regulation	A disease or disorder that affects fewer than 5 in 10,000 citizens is the definition for rare	141/2000	
			EMA	prevalence of rare disease < 5/10 000		
	Germany (1)	1 (1%)		Affect fewer than 1 in 2000 (i.e., a prevalence of 5 or less per 10,000)		
	Latvia (1)	1 (1%)		Affect fewer than 1 in 2000 (i.e., a prevalence of 5 or less per 10,000)		
	Netherlands (1)	1 (1%)		Affect fewer than 1 in 2000 (i.e., a prevalence of 5 or less per 10,000)		
	Poland (2)	2 (2%)		Affect fewer than 1 in 2000 (i.e., a prevalence of 5 or less per 10,000)		

Continent	Country, frequency	# of articles; (%)		(RD) definition	Date	Adopted / developed
	Romania (1)	1 (1%)		Affect fewer than 1 in 2000 (i.e., a prevalence of 5 or less per 10,000)		
	France (2)	2 (2%)		Affect fewer than 1 in 2000 (i.e., a prevalence of 5 or less per 10,000)		
	Ukraine (1)	1 (1%)		Affect fewer than 1 in 2000 (i.e., a prevalence of 5 or less per 10,000)		
Oceania	Australia (10)	10 (11%)		Diseases with a prevalence of 1.1/10 000		
				Diseases with a prevalence < 2000 individuals.		
				Australia have set prevalence's of 1.16 per 100,000 individuals for a given rare disease.		
				Affecting <11/100,000 inhabitants or ,≤2000 Australians		
				Prevalence threshold for orphan disease designation: 0.9 in 10,000		
				The incidence rate is estimated to be 1 in 10,000 individuals for Australia		
	New Zealand (1)	1 (1%)	PHARMAC	Affecting less than 1:50,000 people, which is a considerably lower prevalence threshold than other nations that are from 5 to 76 per 100,000 people		
Asia	Japan (13)	13 (14%)		Japan diseases with a prevalence of 4.0/10,000		
				<50,000 patients in Japan		
				Intractable diseases, is a Japan-specific conception of diseases with (i) unknown etiology (ii) no effective treatment, (iii) rare status (iv) necessity of long-term treatment		
				The incidence rate is estimated to be ≤2.5 cases in 10,000 for Japan		
	Taiwan (7)	7 (8%)	Taiwan Foundation for Rare Disorders	Diseases affecting < 1 in 10,000 that are officially recognized are eligible for medical coverage.	2000	
			Physically and Mentally Disabled Citizens Protection Act	RD is one type of disability	2001	
	China (5)	5 (5%)	the Chinese Society of Genetic Medicine	Genetic disorders affect with less than one over 50,000 of the incidences in Newborn babies.		
				Incidence of the disease in adults or neonates is less than 1 in 500,000 and 1 in 10,000, respectively.		
	South Korea (4)	5 (5%)		Prevalence thresholds have been set at less than 1 per 20,000		
				Prevalence threshold: <4.0 in 10,000		
				< 20,000 people in Korea (i.e., <4 per 10,000 population)		
	Singapore (2)	2 (2%)		Required the disease severity to be life threatening, and severely- or chronically-, debilitating.		
				Prevalence threshold: 37.7 in 10,000		
	India (1)	1 (1%)	ORDI	Threshold for defining a disease as rare if it afflicts 1 in 5,000 individuals		
	Armenian legislation (1)	1 (1%)		There is no specific definition for rare disease only levels of disability which define whether the patient will receive the necessary medicines for free or not		
	Philippines		The DOH, upon recommendation of the RDTWG,			
Africa	Kenya			Required the disease severity to be ,life threatening, and severely- or chronically- ,debilitating.		

Continent	Country, frequency	# of articles; (%)		(RD) definition	Date	Adopted / developed
Eastern Europe & Northern Asia.	Russia (1)	1 (1%)		Maximum prevalence for a rare disease is defined as 1 in 10,000		
South-eastern Europe & Southwestern Asia	Turkey (1)	1 (1%)		Affect no more than 1 in 100,000, which is 50 times less frequent than the European Union definition.		
	WHO (5)	5 (5%)		Rare disease affects at most 6.5 out of every 10,000 individuals.		
				Frequency of 6.5-10/ 10,000 inhabitants		
				Incidence ranges approximately from 0.65-1% in the whole population.		
				Rare disease as affecting 65/100 000~100/100 000 persons.		
	Orphanet, (1)	1 (1%)		Disease inventory, it is evident that the majority of RDs are of genetic etiology, and a smaller percentage is autoimmune or infectious disorders, in addition to some rare cancers."		

The Rare Diseases Act (RDA; the Orphan Drug Act (ODA; the Food and Drug Administration (FDA); The Canadian Organization of Rare Diseases (CORD); National Health Service (NHS); - PHARMAC (the Pharmaceutical Management Agency); *Organization for Rare Diseases India (ORDI)*

Supplementary Table 6: ODs definitions based on continents

Continent	Country, frequency	# of articles; (%)		(RD) definition	Date	Adopted / developed
Europe	EU/UK (22)	19 (20%)	EMA	If the drug is intended for the diagnosis, prevention, or treatment of a life-threatening or chronically and seriously debilitating condition affecting not more than 5 in 10 000 EU people or that it is unlikely that marketing the drug in the EU would generate sufficient benefit for the affected people and for the drug manufacturer to justify the investment		
			NICE	The current NICE appraisal system means orphan drugs that do not meet HST criteria go through the standard technology appraisal (TA) process, with a cost-effectiveness threshold of¬£30 k/QALY, or¬£50 k/QALY when end-of-life criteria are met		
			EURORDIS	Drugs used in the treatment of rare diseases address significant unmet medical needs and are referred to as orphan drugs because, the pharmaceutical industry has little interest under normal market conditions in developing and marketing drugs intended for only a small number of patients suffering from very rare condition.	(2011 c)	

Continent	Country, frequency	# of articles; (%)		(RD) definition	Date	Adopted / developed
			The Orphan Medicinal Product Regulation	Defines OMPs as products for diagnosis, prevention, or treatment of life-threatening or very serious conditions that affect no more than 5 in 10,000 people in the European Union		
			The Netherlands	Defines orphan drug, as either having an official EU orphan designation or if it targets a disease with a prevalence of <1 in 150,000 and shows a clinically proven therapeutic benefit and no other registered medicine exists		
			Poland	There is no specific formal threshold for orphan designations, there is only a general cost-effectiveness threshold that equals 3 x GDP per capita for ICUR/QALY (for CUA) or ICER/LYG (for CEA), which in 2014 is approximately € 26 800.		
	Italian (1)	1 (1%)	Medicines Agency (AIFA)	AIFA may grant a medicine the status of innovative drug according to 3 criteria: unmet medical needs, clinical added value and quality of evidence.		
	German (1)	1 (1%)		Certain special HTA criteria are applied to orphan drugs: Higher P values for small sample sizes; Use of surrogate endpoints, Higher therapeutic benefit is automatically recognised for orphan drugs because these drugs had to prove significant additional therapeutic benefit compared with other possibly already approved drugs as part of the European marketing authorisation procedure. budget impact is less than €50 million per year for a particular indication		
North America	US (9)	8 (9%)	FDA	The defines an OD as 'one intended for the treatment, prevention or diagnosis of a rare disease or condition, which is one that affects less than 200, 000 persons in the USA' (which equates to approximately 6 cases per 10,000 population) 'or meets cost recovery provisions of the act'		
			Orphan Drug Act (ODA)	Orphan drug on the basis of unprofitability: one intended for the diagnosis, treatment, or prevention of a rare disease or condition in the United States, such that there was no reasonable expectation that the costs of developing the drug would be recovered from its sales in the United States. This definition was amended in 1984 to provide, in addition, a prevalence threshold of 200,000 persons affected by the disease. condition of interest in the United States as a surrogate for the lack of profitability."		
				Orphan product, as one that is intended to treat a rare disease or condition that affects fewer than 200,000 people in the United States OR as a product which will not be profitable within seven years of approval by the FDA		
Asia	Singapore (1)	1(1%)	Orphan Drugs Policy	Allows patients with life-threatening and severely debilitating diseases with no other treatment options to access approved drugs prescribed by their practitioner.	1991	
	Korea (2)	2 (2%)	the Orphan Drug Centre	Supplies medicines for diseases affecting fewer than 1 in 20,000.		
			the Korea Ministry of Food and Drug Safety formulates ODs	Drugs used for a disease with 20,000 or fewer patients (population with the disease) and diseases for which adequate treatments or drugs have not yet been developed, or drugs that significantly improve safety or efficacy compared to existing alternatives, are designated as OD		
	China (2)	2 (2%)		Orphan drugs are defined by their availability as pharmaceutical products or active ingredients not developed, imported, or registered owing to low commercial returns and unfavorable marketing conditions.		
				Drug used for diseases affecting fewer than 1 in 10,000		
	Vietnam (1)	1(1%)		Orphan drugs are defined by their availability as pharmaceutical products or active ingredients not developed, imported, or registered owing to low commercial returns and unfavorable marketing conditions		

Supplementary Table 7: URDs definitions based on continents

Continent	Country, frequency	# of articles; (%)		(URD) definition	Date	Adopted / developed
Europe	UK			Ultra-orphan diseases, the term refers to chronic diseases with a prevalence of 1 in 50,000 of the population (Hugheset al., 2005)		
			NICE	Ultra-orphan diseases affect a very small patient population, defined by the National Institute for Health and Care Excellence (NICE) as those diseases with a prevalence of ≤ 1: 50,000		
	Alberta		NICE	URD: conditions with a prevalence of less than 1 per 50,000 persons (NICE, Alberta).		
	England		Advisory Group on National Specialized Services (AGNSS).	The qualifier required by AGNSS was less than 500 persons affected in England (i.e., ~1 in 100,000 of the English population)		
	Ontario			An incidence rate of fewer than 1 in 150,000 live births or new diagnoses per year in Ontario		
				ultra-orphan diseases affecting <1/50000 inhabitants		
			(EU regulation 536/2014)	Ultra-rare diseases have a prevalence of 1 in 50,000 individuals or less in Europe		
	England and Wales		NICE	"Ultra-orphan conditions are defined as diseases affecting <1000 people in England and Wales by the National Institute for Health and Care Excellence (NICE)"		
	Poland			Poland uses the EU definition of 'Ultra-rare being <1 in 50000 people'		Adopted EU definition
				rare disease there are "singular cases" or "individual cases", which are considered "ultra-rare diseases" (prevalence: <1:10,000), including, for example MuSK-positive myasthenia gravis (prevalence 0.05–0.65/100,000 or congenital myasthenic syndrome (CMS)		
				ultra-rare diseases (affecting <20/million persons)"		
				the prevalence can be much lower, leading to the concept of the ‚ultra-orphan disease‚ for diseases with an estimated prevalence of <1 in 50,000 people "		
				Ultra-rare, affecting less than 1 person per 50,000 inhabitants."		

Continent	Country, frequency	# of articles; (%)		(URD) definition	Date	Adopted / developed
				ultra-orphan (prevalence: <1:50,000)		
			NICE Highly Specialised Technology Programme (HSTP) and the SMC	The NICE Highly Specialised Technology Programme (HSTP) and the SMC consider ultra-orphan to be <1 in 50,000 and meeting other specialised criteria. "		

Supplementary Table 8: UODs definitions based on continents

Continent	Country, frequency	# of articles; (%)		(UOD) definition	Date	Adopted / developed
				Ultra-Orphan Drug define as drug for diseases with a prevalence of 0.18/10 000 or less		
				NICE: applied it to drugs with indications for conditions with a prevalence of less than 1 per 50,000 persons"		
				Indications approved for use in diseases with a prevalence of less than 1000 patients (i.e.: ultra-orphan drugs)		
				Definitions of orphan (prevalence ≤5:10,000) and ultra-orphan drug (prevalence ≤1:50,000) were consistent in most countries.		
	Scotland		The Scottish government	new definition of ultra-orphan medicines that can treat very rare conditions affecting fewer than 1 in 50,000 people—approximately 100 people or fewer in Scotland		
	England			HST for ultra-orphan indications Euro113,900-341,700/QALY in England		
			WHO	WHO recommends a WTP of <3 times GDP per capita/QALY		
	Scotland			New definition for ultra-orphan drugs: ‚medicines that are used to treat a condition with a prevalence of 1 in 50,000 or less or around 100 people in Scotland, which will mostly be used to facilitate early access programs and reimbursement processes	Effective from October 2018	
			NICE	No official definition of ‚ultra-orphan disorders‚ has yet been adopted globally. Rather, this informal subcategory was introduced by the National Institute for Health and Care Excellence (formerly, the Institute for Health and Clinical Excellence, and the Institute for Clinical Excellence; NICE), who applied it to drugs with indications for conditions with a prevalence of less than 1 per 50,000 persons"		

Continent	Country, frequency	# of articles; (%)		(UOD) definition	Date	Adopted / developed
			NICE	Currently, no official definition of “ultra-orphan disorders” has been adopted globally. This informal subcategory was introduced by the National Institute for Health and Care Excellence (NICE), which applied it to drugs with indications for conditions with a prevalence of less than 1 per 50,000 persons.		
