# Supplementary material for: Global insight into rare disease and orphan drug definitions: a systematic literature review
Source: BMJ Open. 2025 Jan 25;15(1):e086527. doi: 10.1136/bmjopen-2024-086527 (PMC11784410; doi:10.1136/bmjopen-2024-086527)
Supplement: online supplemental file 6 [file bmjopen-15-1-s006.pdf]

Supplementary Table 9: Qualitative and Quantitative descriptors and themes

RDs Qualitative and Quantitative descriptors and themes

Themes	Qualitative Descriptors
Nature	1. Disease
	2. Condition
	3. Disorder
	4. Pathologies
	5. Status
	6. Severe
	7. Chronic
	8. Serious
	9. Intractable
	10. High Complexity
	11. Medic* (medical, Medicinal, Medically, & Medicine)
	12. Drugs
	13. Heterogeneous Group
Etiology	14. Unknown Etiology
	15. Genetic
	16. Hereditary
**Quantitative Descriptors**	
Measures	1. Prevalence
	2. Absolute # of patients
	3. Incidence
	4. Incidence rate
	5. Frequency
	6. Number of case references
	7. Threshold
	8. Range
	9. Percentage
	10. Estimated measure

Theme	Qualitative Descriptors
Disease nature affecting the pt.	17. Rare
	18. Disable
	19. Life-Limiting condition
	20. Life-threatening
	21. Substantial cause for early death
	22. Long-Term Treatment
	23. Debilitating
Disease nature affecting the pt.'s Society	24. Considerable reduction in an individual's quality of life
	25. Considerable reduction in socio-economic potential
	26. Unmet medical needs
Population characteristics	27. Low Prevalence
	28. Small number of patients
	29. Low Occurrence
	30. Rarely afflict the population
	31. Population
	32. People
	33. Inhabitant* (s)
Indication	34. Treat* (Treatment)
	35. Prevent* (Prevention)

ODs Qualitative and Quantitative descriptors and themes

Themes	Qualitative Descriptors	Themes	Qualitative Descriptors
Nature of Product	1. Medical Product	Unmet Need	21. No alternative treatment
	2. Agent		22. Treatment Price
	3. Biological Products		23. Lack profit

	4. Product		24. Lack of drug development
	5. Pharmaceutical Product		25. Little interest
	6. Active Ingredients not developed, imported, or registered		26. No/limited available therapy
	7. Drug		27. Attractive for commercial development
Disease nature affecting the pt.'s Society.	8. Rare Diseases	Benefits from taking the treatments	28. Clinical added value
	9. Life-Threatening Condition		29. Improve safety or efficacy
	10. Debilitating Disease		30. Product will be of significant benefit
	11. Disease with a limited number of specialist treatment centers		31. New drug is significantly better than drugs currently marketed
	12. Serious Condition	Indication	32. Indications
	13. Rare medical condition		33. Diagnosis
	14. Interactable diseases		34. Treatment
	15. Unmet medical needs		35. Prevention
	16. Common disease where the sponsor cannot make any profit		36. Prophylaxis
			37. Rehabilitation
Population Characteristics	17. Low prevalence		
	18. Small number of patients		
	19. Population		
	20. People		
Quantitative Descriptors			
Measures	1. Prevalence		
	2. Cost-effectiveness threshold		
	3. Annual budget impact for a particular indication		
	4. Number of cases reference		
	5. Willingness to pay (WTP) of <3 times gross domestic product (GDP) per capita/QALY		

URDs Qualitative and Quantitative descriptors and themes

Theme	Qualitative	Theme	Quantitative
**Nature**	1. Disease	**Measurements**	1. Prevalence
	2. Chronic		2. Incidence
**Population Characteristics**	Very small patient		3. Incidence rate
	Population		4. Estimated measure
	People		
	Persons		
	Inhabitants		

UODs Qualitative and Quantitative descriptors and themes

Theme	Qualitative	Theme	Qualitative
Nature	1. Very rare conditions	Indication	1. Indications
	2. Medicines		2. Treat
	3. Drug		3. Approved for use
	4. Disease	Population Characteristics	1. Patients
	5. Condition		2. Persons
**Theme**	**Quantitative**		3. People
Measurements	1. Prevalence		
	2. Willingness to pay (WTP) of <3 times gross domestic product (GDP) per capita/QALY.		

Supplementary Table 10: Qualitative criteria frequently used for RDs, ODs, URDs, and ODs in the definition.

Theme	Qualitative Descriptor	RD	URD	OD	UODs
Nature	1. Disease	148	13	60	2
	2. Condition	30	3	52	4
	3. Disorder	18	1	2	1
	4. Pathologies	1	-	1	-
	5. Status	1	-	2	-
	6. Sever*	5	-	5	-
	7. Chronic	22	1	7	-
	8. Serious	3	-	12	-
	9. Intractable	1	-	1	-
	10. High Complexity	1	-	-	-
	11. Heterogeneous	1	-	-	-
	12. Product	-	-	35	-
	13. Medic* (medical, Medicinal, Medically, & Medicine)	5	-	36	2
	14. Agent	-	-	1	-
	15. Biological Products	-	-	1	-
	16. Pharmaceutical Product	-	-	2	-
	17. Active Ingredient not developed, imported, or registered	-	-	1	-
	18. Drugs	8	-	83	8
Etiology	19. Unknown Etiology	1	-	-	-
	20. Genetic	7	-	1	-
	21. Hereditary	1	-	-	-
Diseas e nature affecti [illegible]	22. Rare Diseases	40	4	16	-
	23. Disab* (Disability & Disabling)	5	-	2	-
	24. Life -Limiting	1	-	0	-

Theme	Qualitative Descriptor	RD	URD	OD	UODs
	25. Life-threatening	23	-	20	-
	26. Substantial cause for early death	1	-	0	-
	27. Long-Term Treatment	1	-	0	-
	28. Debilitating	21	-	10	-
Disease nature affecting the pt.'s Society	29. Considerable reduction in an individual's quality of life	1	-	0	-
	30. Considerable reduction in socio- economic potential	2	-	0	-
	31. Unmet medical needs	3	-	3	-
	32. Disease with limited number of specialist treatment centers	-	-	1	-
	33. Common disease where the sponsor cannot make any profit	-	-	1	-
Population Characteristics	34. Low Prevalence	12	-	2	-
	35. Low Occurrence	2	-	-	-
	36. Rarely afflict the population	1	-	-	-
	37. Small number of patients	3	-	1	-
	38. Very small patient Population	-	1	-	-
	39. Population	20	3	7	-
	40. People	29	2	8	2
	41. Inhabitant* (s)	6	2	-	-
Benefits from taking the treatment	42. Clinical added value	-	-	1	-
	43. Improve safety or efficacy	-	-	1	-
	44. Product will be of significant benefit	-	-	2	-
	45. New drug is significantly better than drugs currently marketed	-	-	1	-
Indication	46. Indications	-	-	4	4
	47. Diagnosis	-	-	23	-
	48. Treat* (Treatment)	7	-	55	2
	49. Prevent* (Prevention)	1	-	23	-
	50. Rehabilitation	-	-	1	-
	51. Prophylaxis	-	-	1	-

Supplementary Table 11: Quantitative criteria frequency used of RDs, ODs, URDs, and ODs in the definition.

Theme	Quantitative Descriptor	RD	URD	OD	UOD
Measurements	1. Prevalence	51	10	22	6
	2. Absolute # of patients	1	-	-	-
	3. Incidence	7	1	-	-
	4. Incidence rate	2	1	-	-
	5. Frequency	1	-	-	-
	6. Number of* (cases reference, patients, people, prevalent cases, and individuals)	6	-	5	-

	7. Threshold	3	-	-	-
	8. Estimated measure	5	1	-	-
	9. Range	2	-	-	-
	10. Percentage	3	-	-	-
	11. Cost-effectiveness threshold	-	-	2	-
	12. Annual budget impact for a particular indication	-	-	1	-
	13. willingness to pay (WTP) of <3 times gross domestic product (GDP) per capita/QALY	-	-	1	1
