# Supplementary material for: Global insight into rare disease and orphan drug definitions: a systematic literature review
Source: BMJ Open. 2025 Jan 25;15(1):e086527. doi: 10.1136/bmjopen-2024-086527 (PMC11784410; doi:10.1136/bmjopen-2024-086527)
Supplement: Supplementary file 7 [file bmjopen-2024-086527.reviewer_comments.pdf]

PEER REVIEW HISTORY

BMJ Open publishes all reviews undertaken for accepted manuscripts. Reviewers are asked to complete a checklist review form and are provided with free text boxes to elaborate on their assessment. These free text comments are reproduced below.

ARTICLE DETAILS

Title (Provisional)

Global Insight into Rare Disease and Orphan Drug Definitions: A Systematic Literature Review

Authors

Abozaid, Ghada Mohammed; Kerr, Katie; Alomary, Hiba; Al-Omar, Hussain A.; McKnight, Amy

---

VERSION 1 - REVIEW

**Reviewer**	**1**
**Name**	**Giannuzzi, Viviana**
**Affiliation**	**Fondazione per la Ricerca Farmacologica Gianni Benzi ONLUS**
**Date**	**16-Apr-2024**
**COI**	**none**

Rare diseases and orphan drugs represent a biomedical field where standardisation is of utmost importance. Therefore, papers discussing on this topic are more than welcome. Here below some comments to improve the manuscript.

First of all, a work focused on definitions should not be based only on literature search because the real definitions come from the regulations, national laws, inetrnational guidelines, i.e. from the regulatory framework. In fact, the definitions provided by the authors themselves in the results come from the regulatory frameworks.

In addition, results do not report the references of articles retrieved from the literature and supporting definitions.

This aspect is not mentioned in the "Strengths and limitations"

Other comments.

- line 46: the definition of ‘orphan disease’ is available and should be provided (with appropriate reference)

- Inconsistencies among definitions have been already discussed also in 2017 (doi: 10.1186/s13023-017-0617-1)

- the INTRODUCTION could mention why harmonisation is needed in the frare diseases field, and therefore which are the challenges mentioned in line 84

- the authors should explain why rare cancers have been excluded by the analysis (line 103)

- the authors should better explain the methodological differences (if any) between the present analysis and the protocol "The protocol for this SLR" published 2 years ago to BMJ open: https://bmjopen.bmj.com/content/bmjopen/12/7/e062126.full.pdf (Ref n 9 of this manuscript).

**Reviewer**	**2**
**Name**	**Vasilevsky, Nicole**
**Affiliation**	**Data Collaboration Center, Critical Path Institute**
**Date**	**29-May-2024**
**COI**	**None**

This paper describes a systematic review to determine definitions of rare disease concepts, orphan drugs, and their subtypes by performing a search of major publication databases. This paper is very relevant to the scientific and medical community, because, as the authors mentioned, providing clear and concise definitions of rare diseases will help improve diagnoses, and access to treatment and promote research.

The point about country-specific definitions is very valid and I agree that creating country-specific definitions would indeed be very helpful.

There is a typo on line 33, RDS -> RDs

Another relevant publication for reference 1 is: https://www.ncbi.nlm.nih.gov/pmc/articles/PMC7771654/

The supplemental table that includes all of the rare disease definitions is helpful. 1) it would be nice to share this table as a downloadable csv file (if the journal doesn't already do that, it could be shared in FigShare or a similar data repository); 2) it would be nice to include a summary table in the main text that summarizes the content in the rare disease definitions by country section. This could be done for the URDs and others too.

One thing that is missing from this paper is a discussion of data standards that aim to formally define rare disease concepts. Many controlled terminologies and ontologies exist that seek to do just that - provide formal and structured definitions of rare diseases. Some examples include:

- OMIM (Mendelian diseases)
- Orphanet (rare diseases in Europe)
- NORD
- GARD
- Mondo Disease Ontology
- Disease Ontology

These terminologies do not necessarily define the term 'rare disease' itself, but provide definitions and classifications of known rare disease concepts that are subtypes of rare disease. It is worth mentioning these in this paper because they are formal vocabularies that aim to provide definitions and consensus amongst the community.

**Reviewer**	**3**
**Name**	**Robinson, Peter**
**Affiliation**	**The Jackson Laboratory for Genomic Medicine**
**Date**	**07-Sep-2024**
**COI**	**none**

The authors provide a systematic literature review on the definition of “rare disease” and related concepts. There is no universally agreed upon definition of an RD, for instance the definitions in the EU and the US differ somewhat.

The authors provide a Systematic Literature Review with a summary of definitions about RDs in many countries as derived from published sources.

There have been a number of review articles on this topic previously. The authors should provide a more comprehensive description of existing reviews on the topic in their introduction. The authors should more clearly state the deficit in the previous literature they were trying to address with this project, and in the discussion they should review what additional benefit their study provides.

There are well known differences in definitions; for instance, the USA, a RD is defined as one that affects fewer than 200,000 Americans, while in Europe and many other countries, a RD is defined as one that affects fewer than 1 in 2000 individuals.

It would be interesting to hear about what the actual conseuqneces of these differences have been in the past decades. The authors write that “subjective standards may result in inconsistent results and implementation challenges. For comprehensive definitions of RDs, ODs, and their subtypes, it is better to combine qualitative and quantitative criteria to be reviewed and updated periodically.” While this seems plausible, so many factors go into the quality of health care and research on RDs, that it is not obvious that the definition of what an RD is actually is a major factor. For instance, the health care systems in USA and Europe are vastly different, which has major consequences for individuals with RDs. On the other hand, I do not know of cases where a specific disease is called “rare” in the US but not in Europe (or vice versa), and where this has led to specific problems for individuals affected by that disease – it would add a lot of interest to the paper if the authors could summarize evidence about this and provide literature references.

The author chose to exclude non-genetic RDs (cancer, infections, poisoning), but there is no obvious reason for doing so, since these individuals will experience many of the same problems. The authors should make it clear that they have investigated genetic RDs or should consider describing the differences between genetic and non-genetic RDs.

VERSION 1 - AUTHOR RESPONSE

Reviewer: 1

Dr. Viviana Giannuzzi, Fondazione per la Ricerca Farmacologica Gianni Benzi ONLUS.

Comments to the Author:

1. First of all, a work focused on definitions should not be based only on literature search because the real definitions come from the regulations, national laws, inetrnational guidelines, i.e. from the regulatory framework. In fact, the definitions provided by the authors themselves in the results come from the regulatory frameworks. In addition, results do not report the references of articles retrieved from the literature and supporting definitions. This aspect is not mentioned in the "Strengths and limitations"
2. line 46: the definition of 'orphan disease' is available and should be provided (with appropriate reference).
3. Inconsistencies among definitions have been already discussed also in 2017 (doi: 10.1186/s13023-017-0617-1).
4. the INTRODUCTION could mention why harmonisation is needed in the frare diseases field, and therefore which are the challenges mentioned in line 84.
5. the authors should explain why rare cancers have been excluded by the analysis (line 103).
6. the authors should better explain the methodological differences (if any) between the present analysis and the protocol "The protocol for this SLR" published 2 years ago to BMJ open: https://bmjopen.bmj.com/content/bmjopen/12/7/e062126.full.pdf (Ref n 9 of this manuscript).

__Comment 1__: ***[First of all, a work focused on definitions should not be based only on literature search because the real definitions come from the regulations, national laws, international guidelines, i.e. from the regulatory framework. In fact, the definitions provided by the authors themselves in the results come from the regulatory frameworks. In addition, results do not report the references of articles retrieved from the literature and supporting definitions. This aspect is not mentioned in the "Strengths and limitations"]***

__**Response:**__ We appreciate Dr. Giannuzzi's feedback regarding the focus on definitions and the role of regulatory frameworks. However, we respectfully disagree with the suggestion that this aspect is not mentioned in the "Strengths and limitations" section. In this section, we explicitly acknowledge that the review explores different criteria for defining RDs and

ODs issued by various agencies and entities to fulfil their mandates (second bullet point). These agencies operate within the context of regulatory frameworks, which are integral to the definitions provided. We believe this sufficiently reflects the role of regulations and guidelines in shaping RD and OD definitions. Additionally, we have ensured that the definitions cited in the results section are properly linked to the relevant regulatory frameworks, as noted in the updated manuscript.

Comment 2: ***[line 46: the definition of 'orphan disease' is available and should be provided (with appropriate reference).]***

Response: Thank you for your insightful feedback. In response to your suggestion to include a definition of "orphan disease," we have added the appropriate references. Orphan diseases, often synonymous with rare diseases, are those that affect a small percentage of the population and lack sufficient market potential for the pharmaceutical industry to invest in developing treatments. Aronson (2006) defines orphan diseases as a term has been used to denote neglected diseases. Additionally, Fehr and Prütz (2023) emphasize that rare diseases are also referred to as 'orphan diseases'. We have made the requested changes in the manuscript by adding the refernces and **highlighted them in red** for easy reference. And it present between **437 – 439 lines**.

Changes: We have now incorporated these definitions and references into the manuscript to enhance clarity on the terminology:

- [1]Aronson J. Rare diseases, orphan drugs, and orphan diseases. BMJ. 2006;333:127-128.
- [2]Fehr A, Prütz F. Rare diseases: a challenge for medicine and public health. Journal of health monitoring. 2023;8:3-6.

Comment 3: ***[Inconsistencies among definitions have been already discussed also in 2017 (doi: 10.1186/s13023-017-0617-1).]***

Response: We understand Dr. Viviana Giannuzzi's observation about the prior discussion of inconsistencies in the definitions of RDs and ODs, which was explored in Giannuzzi et al.'s article, "Orphan Medicinal Products in Europe and the United States to Cover Needs of Patients with Rare Diseases." We acknowledge the importance of this foundational work, which thoroughly examines regulatory challenges within the EU and US frameworks and emphasizes collaboration as a route to improved access. Our study, however, was designed to build upon and extend these discussions by incorporating a more comprehensive and globally orientated analysis of RD and OD definitions to build a foundational understanding of definitions worldwide. Both studies aim to tackle the difficulties and discrepancies in defining RDs and ODs, their influence on healthcare accessibility, drug availability, and regulatory procedures, through a methodical approach to data collection. In addition, both studies identify how differing definitions and prevalence thresholds impact RD and OD designations, with each analysis emphasizing the significant consequences of these variations for patient care and healthcare policies. But there are differences between both studies. We will highlight the distinctions and contributions of our work, "Global Insight into Rare Disease and Orphan Drug Definitions: A Systematic Literature Review."

- **Aim and Scope:** Our study provides a **global perspective** on rare disease (RD) and orphan drug (OD) definitions, whereas Giannuzzi et al. focus specifically on the **EU and US regulatory** frameworks.
- **Methodological Approach**: Our work, driven by a **systematic literature review** across multiple databases (Medline, EMBASE, Scopus, and Web of Science) and adhering to PRISMA-P and PROSPERO guidelines, with detailed protocol registered with PROSPERO, identifies over 200 distinct definitions of RDs and ODs across various regions. This approach allows us to analyse prevalence-based, qualitative, and quantitative criteria, highlighting how regional definitions impact healthcare accessibility, policy, and drug development in broader geographic contexts. In contrast, Giannuzzi et al. examine **data directly from FDA and EU orphan drug registries,** collects data on approved drugs, therapeutic indications, target population, and genetic conditions, to assess how existing frameworks and incentives can improve access to orphan drugs within the EU and US, recommending collaboration as a path to expanded access.

- Our findings **underscore the importance of tailored, regionally adapted definitions** to better address genetic, societal, and environmental factors influencing rare disease prevalence worldwide, supporting the development of a globally relevant framework for RD and OD designation. While the Giannuzzi et al study **discussed inconsistencies within the EU and US frameworks** and suggested regulatory collaboration to mitigate these.
- **Policy Implications:** Our manuscript emphasizes the need for both universal and regionally adaptable definitions to promote consistent healthcare policies and improve access to treatments for rare diseases. This policy-focused approach is distinct from Giannuzzi et al.'s work, which, while supportive of increased EU-US regulatory collaboration, does not advocate for specific country-level definition adjustments outside of these regions.

Comment 4: ***[the INTRODUCTION could mention why harmonisation is needed in the rare diseases field, and therefore which are the challenges mentioned in line 84.]***

Response: Thank you for your valuable comment regarding the need for harmonization in the rare diseases field. In response, we have expanded the Introduction to emphasize why harmonization is critical and to outline the key challenges associated with the current lack of uniformity in definitions. We have made the requested changes in the manuscript by adding the refernces and **highlighted them in red** for easy reference. And it present between **84 – 97 lines**.

Changes: There are considerable challenges associated with the context and practical use of RDs, ODs, and subtype definitions employed by various stakeholders. One significant challenge is the inconsistency in definitions across regions and regulatory agencies. For example, the EU and the US use different prevalence thresholds to define RDs, complicating regulatory frameworks and market access for ODs. This variation also affects clinical trials and research, as the lack of harmonized definitions can hinder data comparability and international collaboration. Moreover, pharmaceutical companies face additional regulatory and pricing barriers due to these differences, which can delay drug approval and patient access. From a patient care perspective, disparities in definitions may lead to inequities in diagnosis, treatment, and access to therapies. OD treatments may not be available to patients

in other regions with the same condition, fragmenting advocacy efforts. Finally, economic and ethical considerations, such as cost-effectiveness criteria and the financial burden on healthcare systems, further complicate the practical use of the RD and OD definitions, highlighting the need for harmonization to ensure equitable and efficient healthcare delivery globally for RD patients.

Comment 5: ***[the authors should explain why rare cancers have been excluded by the analysis (line 103).]***

Response: In response to your comment regarding the exclusion of rare cancers from the analysis, we recognise that rare cancers are a critical aspect of RDs. However, for the purposes of this systematic review, we chose to exclude rare cancers to maintain a focused scope and ensure that the analysis remains manageable and relevant to the broader definitions of RDs and ODs. Rare cancers often have distinct clinical, regulatory, and research considerations compared to non-cancerous RDs. For instance, they typically fall under oncology-specific frameworks that include well-established criteria for diagnosis, treatment pathways, and regulatory incentives like OD designation. Adding rare cancers would have made things more complicated and might have taken away from the more important issues surrounding the definition and regulation of RDs that aren't cancer and ODs. Furthermore, previous literature and regulatory frameworks often treat rare cancers separately, and thus, their exclusion allows us to provide a more cohesive analysis of RDs and ODs that are not cancer-specific. Therefore, we made a deliberate methodological choice to exclude rare cancers in order to produce clear and actionable findings within the intended scope of this review. In addtion, in the SLR published protocol I elaborated on the rationale for focusing on genetically RDs. I added the protocol reference in **line 116** and **highlighted red.**

Changes: Added the protocol reference [3]

Comment 6: ***[the authors should better explain the methodological differences (if any) between the present analysis and the protocol "The protocol for this SLR" published 2 years ago to BMJ open:***

https://bmjopen.bmj.com/content/bmjopen/12/7/e062126.full.pdf (Ref n 9 of this manuscript).]

Response: In response to your comment regarding the need to clarify methodological differences between the current analysis and the previously published protocol. The methodological foundation of this SLR remains consistent with the BMJ Open protocol, adhering to the principles of systematic review design based on PRISMA-P guidelines and registration with PROSPERO. However, there are some minor methodological refinements made during the execution phase. For instance, updated searches were performed on 31st December 2022 and 31st December 2023 to ensure the most recent studies were captured. This allowed the review to include more contemporary studies and ensure comprehensive coverage of literature post-dating the protocol publication.

Reviewer: 2

Dr. Nicole Vasilevsky, Data Collaboration Center, Critical Path Institute.

Comments to the Author:

1. There is a typo on line 33, RDS -> RDs
2. Another relevant publication for reference 1 is: https://www.ncbi.nlm.nih.gov/pmc/articles/PMC7771654/
3. The supplemental table that includes all of the rare disease definitions is helpful.
   a) it would be nice to share this table as a downloadable csv file (if the journal doesn't already do that, it could be shared in FigShare or a similar data repository).
   b) it would be nice to include a summary table in the main text that summarizes the content in the rare disease definitions by country section. This could be done for the URDs and others too.
4. One thing that is missing from this paper is a discussion of data standards that aim to formally define rare disease concepts. Many controlled terminologies and ontologies exist that seek to do just that - provide formal and structured definitions of rare diseases. Some examples include: OMIM (Mendelian diseases), Orphanet (rare diseases in Europe), NORD, GARD, Mondo Disease Ontology, and Disease Ontology. These terminologies do not necessarily define the term 'rare disease' itself but provide definitions and classifications of known rare disease concepts that are subtypes of rare disease. It is worth mentioning these in this paper because they are formal vocabularies that aim to provide definitions and consensus amongst the community.

Comment 1: ***[There is a typo on line 33, RDS -> RDs]***

Response: In response to your comment regarding the typographical error on line 33, which refers to "RDS" instead of "RDs," we appreciate the attention to detail. I corrected this error in the final version of the manuscript to ensure accuracy and clarity. We have made the requested changes in the manuscript by adding the refernces and **highlighted them in red** for easy reference, and it present in **line 37.**

Comment 2: ***[Another relevant publication for reference 1 is:***

https://www.ncbi.nlm.nih.gov/pmc/articles/PMC7771654/

Response: In response to Dr. Nicole Vasilevsky's comment, our study aligns with Haendel et al.'s article, "How many rare diseases are there?" Both studies address the inconsistencies in rare disease (RD) definitions and emphasize the need for clearer frameworks, which have an impact on healthcare access, regulatory policies, RD designations, and patient outcomes. Both studies emphasize the need for consistent definitions, with Haendel et al. advocating terminology harmonization and our study proposing a dual approach to global standardization and region-specific criteria. While Haendel et al. focus on accurately quantifying the number of RDs and the necessary terminological harmonization to achieve this goal, our manuscript offers a broader exploration of global definitions for both RDs and ODs, as well as the availability of orphan drugs and their policy implications. The two studies differ in several aspects:

- The aim of our study is to provide a comprehensive global review of definitions and criteria for rare diseases (RDs) and orphan drugs (ODs), examining over 200 definitions from various regulatory and healthcare contexts. The Haendel et al. article endeavours to measure the quantity of rare diseases by analysing disease terminologies from various databases.
- Our study focuses on understanding the challenges and implications of definitional inconsistencies for healthcare policies, drug development, and patient access. We suggest a more equitable structure that takes into account both global definitions and regional modifications to enhance healthcare results. The Haendel et al. study emphasizes difficulties in estimating RD counts due to inconsistent terminologies and a lack of standard definitions, calling for a global consensus to improve precision in RD classification and treatment strategies.
- Our methodology involves conducting a systematic literature review using databases such as Medline, EMBASE, Scopus, and Web of Science, with a focus on extracting definitions and criteria for RDs and ODs. This approach includes a broad range of sources to identify qualitative and quantitative criteria used in various regions and healthcare frameworks. The second study employs a computational approach, analyzing disease terminology databases such as Orphanet, OMIM, and GARD, with a

focus on ontological and terminological aspects to estimate the number of distinct RDs. The methodology emphasizes hierarchical structuring and the ontology of disease terms to calculate the number of RDs accurately and discuss the disparities in counts across sources.

- Our study identifies extensive variability in RD and OD definitions globally, noting that criteria based solely on prevalence are insufficient due to differing population structures and healthcare needs. The study advocates for region-specific definitions to address local epidemiological factors while encouraging some degree of global standardization. By consolidating disease terminologies across databases, Haendel et al. estimate 10,000 RDs, revealing a higher number than traditionally assumed. Additionally, the study addresses the fundamental challenges in counting and categorizing RDs across international databases. The increasing difficulties in accurately diagnosing and treating rare diseases necessitate the harmonisation of terminology.

Comment 3.a: ***[The supplemental table that includes all of the rare disease definitions is helpful. a) it would be nice to share this table as a downloadable csv file (if the journal doesn't already do that, it could be shared in FigShare or a similar data repository)]***

Response: Thank you for your suggestion regarding the supplemental table of RDs, OD, and their subtypes definitions. We appreciate your input and would like to inform you that the tables has been submitted as a supplemental files to BMJ Open.

Comment 3.b: ***[The supplemental table that includes all of the rare disease definitions is helpful. b) it would be nice to include a summary table in the main text that summarizes the content in the rare disease definitions by country section. This could be done for the URDs and others too.]***

Response: Thank you for your suggestion to include a summary table within the main text. In response, we would like to highlight that four comprehensive tables summarizing RDs, ODs, and their subtypes based on definitions from various countries have already been included in the manuscript. These tables provide an overview of the definitions as used by

different regulatory authorities and institutions globally, which we believe aligns with your recommendation. We have made the requested changes in the manuscript by adding the refernces and **highlighted them in red** for easy reference, and it present in between **lines 239- 243, 256- 257, 283 -284, and 296 - 298**

Changes:

Table 1: A summary of RDs definitions is provided based on the country

Country, frequency	# of articles; (%)		(RD) definition	Date
US (25)	24 (26%)	Orphan Drug Regulation	Defines RD according to prevalence: "rare disease' means any disease or condition that affects less than 200000 persons in the USA'.	1993
		RDA		2002
		ODA	Defined RDs based on qualitative descriptors as follows: 'the term 'rare disease or condition' means any disease or condition which occurs so infrequently in the USA that there is no reasonable expectation that the cost of developing and making available in the USA a drug for such disease or condition will be recovered from sales in the USA of such drug'.	1983
		FDA	Define RD as 'any disease or condition that affects less than 200000 people in the USA or affects >200000 in the USA and for which there is no reasonable expectation that the cost of developing and making available in the USA a drug for such disease or condition will be recovered from sales in the USA of such drug'	
Canada (3)	2 (2%)	CORD	Rare disease as one that afflicts less than 1 person in 200 000.	
			Estimated that 1 in 12 Canadians, or about 2.8 million individuals, may be living with a rare disease	
UK (3)	2 (2%)	the Rare Disease Framework	Defined RD based on prevalence, as a condition affecting fewer than 1 in 2000 people. (i.e., a prevalence of 5 or less per 10,000)	2021
		NHS	Some countries use additional definitions in situations where a condition is not officially defined as rare. classifies all conditions that require specialized medical care as rare if they occur in <500 citizens yearly	
EU (36)	35 (38%)		Rare diseases, including those of genetic origin, are life-threatening or chronically debilitating diseases which are of such low prevalence (less than 5 per 10,000persons in the European Union) that special combined efforts are needed to address them so as to prevent significant morbidity or perinatal or early mortality or a considerable reduction in an individual's quality of life or socio-economic potential.	
		European Commission on Public Health	Defines rare diseases as ,life-threatening or chronically debilitating diseases which are of such low prevalence that special combined efforts are needed to address them.	
		Orphan Drug Regulation	A disease or disorder that affects fewer than 5 in 10,000 citizens is the definition for rare	141/2000
		EMA	prevalence of rare disease < 5/10 000	
France (2)	2 (2%)		Affect fewer than 1 in 2000 (i.e., a prevalence of 5 or less per 10,000)	
Japan (13)	13		Japan diseases with a prevalence of 4.0/10,000	

Country, frequency	# of articles; (%)		(RD) definition	Date
	(14%)		<50,000 patients in Japan	
			Intractable diseases, is a Japan-specific conception of diseases with (i) unknown etiology (ii) no effective treatment, (iii) rare status (iv) necessity of long-term treatment	
			The incidence rate is estimated to be ≤2.5 cases in 10,000 for Japan	
Taiwan (7)	7 (8%)	Taiwan Foundation for Rare Disorders	Diseases affecting < 1 in 10,000 that are officially recognized are eligible for medical coverage.	2000
		Physically and Mentally Disabled Citizens Protection Act	RD is one type of disability	2001
China (5)	5 (5%)	the Chinese Society of Genetic Medicine	Genetic disorders affect with less than one over 50,000 of the incidences in Newborn babies.	
			Incidence of the disease in adults or neonates is less than 1 in 500,000 and 1 in 10,000, respectively.	
South Korea (4)	5 (5%)		Prevalence thresholds have been set at less than 1 per 20,000	
			Prevalence threshold: <4.0 in 10,000	
			< 20,000 people in Korea (i.e., <4 per 10,000 population)	
WHO (5)	5 (5%)		Rare disease affects at most 6.5 out of every 10,000 individuals.	
			Frequency of 6.5-10/ 10,000 inhabitants	
			Incidence ranges approximately from 0.65-1% in the whole population.	
			Rare disease as affecting 65/100 000~100/100 000 persons.	
Orphanet, (1)	1 (1%)		Disease inventory, it is evident that the majority of RDs are of genetic etiology, and a smaller percentage is autoimmune or infectious disorders, in addition to some rare cancers."	

The Rare Diseases Act (RDA; the Orphan Drug Act (ODA; the Food and Drug Administration (FDA); The Canadian Organization of Rare Diseases (CORD); National Health Service (NHS).

Table 2: A summary of URDs definitions is provided based on the country.

Country, frequency		(URD) definition
UK		Ultra-orphan diseases, the term refers to chronic diseases with a prevalence of 1 in 50,000 of the population (Hugheset al., 2005)
	NICE	Ultra-orphan diseases affect a very small patient population, defined by the National Institute for Health and Care Excellence (NICE) as those diseases with a prevalence of ≤ 1: 50,000
England	Advisory Group on National Specialized Services (AGNSS).	The qualifier required by AGNSS was less than 500 persons affected in England (i.e., ~1 in 100,000 of the English population)
Ontario		An incidence rate of fewer than 1 in 150,000 live births or new diagnoses per year in Ontario
England and Wales	NICE	"Ultra-orphan conditions are defined as diseases affecting <1000 people in England and Wales by the National Institute for Health and Care Excellence (NICE)"
Poland		Poland uses the EU definition of 'Ultra-rare being <1 in 50000 people'

Table 3: A summary of ODs definitions is provided based on the country.

Country, frequency	# of articles; (%)		(RD) definition	Date
EU/UK (22)	19 (20%)	EMA	If the drug is intended for the diagnosis, prevention, or treatment of a life-threatening or chronically and seriously debilitating condition affecting not more than 5 in 10 000 EU people or that it is unlikely that marketing the drug in the EU would generate sufficient benefit for the affected people and for the drug manufacturer to justify the investment	
		NICE	The current NICE appraisal system means orphan drugs that do not meet HST criteria go through the standard technology appraisal (TA) process, with a cost-effectiveness threshold of¬£30 k/QALY, or¬£50 k/QALY when end-of-life criteria are met	
		EURORDIS	Drugs used in the treatment of rare diseases address significant unmet medical needs and are referred to as orphan drugs because, the pharmaceutical industry has little interest under normal market conditions in developing and marketing drugs intended for only a small number of patients suffering from very rare condition.	(2011c)

Country, frequency	# of articles; (%)		(RD) definition	Date
		The Orphan Medicinal Product Regulation	Defines OMPs as products for diagnosis, prevention, or treatment of life-threatening or very serious conditions that affect no more than 5 in 10,000 people in the European Union	
		The Netherlands	Defines orphan drug, as either having an official EU orphan designation or if it targets a disease with a prevalence of <1 in 150,000 and shows a clinically proven therapeutic benefit and no other registered medicine exists	
		Poland	There is no specific formal threshold for orphan designations, there is only a general cost-effectiveness threshold that equals 3 x GDP per capita for ICUR/QALY (for CUA) or ICER/LYG (for CEA), which in 2014 is approximately € 26 800.	
US (9)	8 (9%)	FDA	The defines an OD as 'one intended for the treatment, prevention or diagnosis of a rare disease or condition, which is one that affects less than 200, 000 persons in the USA' (which equates to approximately 6 cases per 10,000 population) 'or meets cost recovery provisions of the act'	
		Orphan Drug Act (ODA)	Orphan drug on the basis of unprofitability: one intended for the diagnosis, treatment, or prevention of a rare disease or condition in the United States, such that there was no reasonable expectation that the costs of developing the drug would be recovered from its sales in the United States. This definition was amended in 1984 to provide, in addition, a prevalence threshold of 200,000 persons affected by the disease. condition of interest in the United States as a surrogate for the lack of profitability."	
			Orphan product, as one that is intended to treat a rare disease or condition that affects fewer than 200,000 people in the United States OR as a product which will not be profitable within seven years of approval by the FDA	
Korea (2)	2 (2%)	the Orphan Drug Centre	Supplies medicines for diseases affecting fewer than 1 in 20,000.	
		the Korea Ministry of Food and Drug Safety formulates ODs	Drugs used for a disease with 20,000 or fewer patients (population with the disease) and diseases for which adequate treatments or drugs have not yet been developed, or drugs that significantly improve safety or efficacy compared to existing alternatives, are designated as OD	
China (2)	2 (2%)		Orphan drugs are defined by their availability as pharmaceutical products or active ingredients not developed, imported, or registered owing to low commercial returns and unfavorable marketing conditions.	
			Drug used for diseases affecting fewer than 1 in 10,000	

Table 4: A summary of UODs definitions is provided based on the country.

Country, frequency		(UOD) definition	Date
UK	NICE	Drugs with indications for conditions with a prevalence of less than 1 per 50,000 persons"	
Scotland	The Scottish government	new definition of ultra-orphan medicines that can treat very rare conditions affecting fewer than 1 in 50,000 people—approximately 100 people or fewer in Scotland	
England		HST for ultra-orphan indications Euro113,900-341,700/QALY in England	
	WHO	WHO recommends a WTP of <3 times GDP per capita/QALY	
Scotland		New definition for ultra-orphan drugs: ‚medicines that are used to treat a condition with a prevalence of 1 in 50,000 or less or around 100 people in Scotland, which will mostly be used to facilitate early access programs and reimbursement processes	Effective from October 2018

<u>Comment 4:</u> ***[One thing that is missing from this paper is a discussion of data standards that aim to formally define rare disease concepts. Many controlled terminologies and ontologies exist that seek to do just that - provide formal and structured definitions of rare diseases. Some examples include: OMIM (Mendelian diseases), Orphanet (rare diseases in Europe), NORD, GARD, Mondo Disease Ontology, and Disease Ontology. These terminologies do not necessarily define the term 'rare disease' itself but provide definitions and classifications of known rare disease concepts that are subtypes of rare disease. It is worth mentioning these in this paper because they are formal vocabularies that aim to provide definitions and consensus amongst the community.]***

<u>Response:</u> Thank you for your insightful comment. We agree that controlled terminologies and ontologies such as OMIM (for Mendelian diseases), Orphanet (for European rare diseases), NORD, GARD, Mondo Disease Ontology, and Disease Ontology play a critical role in classifying and formalizing rare disease subtypes, even though they do not define the overarching term "rare disease" itself. These data standards play a vital role in ensuring consistency in how RDs are classified and understood across different regions and stakeholders. However, the primary aim of this systematic literature review is to shed light on the available global definitions, classifications, and criteria used for RDs, ODs, and their subtypes and to provide insights into the rationale behind these definitions. Given the focus of our review, we have chosen to center the discussion on the broader global definitions and criteria, and a detailed examination of disease-specific ontologies may be outside the scope of the current analysis.

Reviewer: 3

Prof. Peter Robinson, The Jackson Laboratory for Genomic Medicine

Comments to the Author:

1. There have been a number of review articles on this topic previously. The authors should provide a more comprehensive description of existing reviews on the topic in their introduction. The authors should more clearly state the deficit in the previous literature they were trying to address with this project, and in the discussion, they should review what additional benefit their study provides.
2. There are well known differences in definitions; for instance, the USA, a RD is defined as one that affects fewer than 200,000 Americans, while in Europe and many other countries, a RD is defined as one that affects fewer than 1 in 2000 individuals. It would be interesting to hear about what the actual consequences of these differences have been in the past decades.
3. The authors write that "subjective standards may result in inconsistent results and implementation challenges. For comprehensive definitions of RDs, ODs, and their subtypes, it is better to combine qualitative and quantitative criteria to be reviewed and updated periodically." While this seems plausible, so many factors go into the quality of health care and research on RDs, that it is not obvious that the definition of what an RD is actually is a major factor. For instance, the health care systems in USA and Europe are vastly different, which has major consequences for individuals with RDs. On the other hand, I do not know of cases where a specific disease is called "rare" in the US but not in Europe (or vice versa), and where this has led to specific problems for individuals affected by that disease – it would add a lot of interest to the paper if the authors could summarize evidence about this and provide literature references.
4. The author chose to exclude non-genetic RDs (cancer, infections, poisoning), but there is no obvious reason for doing so, since these individuals will experience many of the same problems. The authors should make it clear that they have investigated genetic RDs or should consider describing the differences between genetic and non-genetic RDs.

Comment 1: ***[There have been a number of review articles on this topic previously. The authors should provide a more comprehensive description of existing reviews on the topic in their introduction. The authors should more clearly state the deficit in the previous literature they were trying to address with this project, and in the discussion, they should review what additional benefit their study provides.]***

Response: We appreciate Prof. Peter's feedback and the opportunity to clarify our study's contributions relative to existing literature. The table below captures the distinct approaches and focuses across several studies.

Our manuscript represents a unique and comprehensive contribution to the field. While prior studies, such as those by Haendel et al. and Giannuzzi et al., have addressed aspects of rare disease (RD) and orphan drug (OD) definitions, they are either region-specific or focus on narrow elements like database discrepancies or regulatory practices in specific regions (e.g., EU and US). our study is distinct in its global approach, systematically reviewing over 200 definitions from diverse geographic and regulatory contexts. By comparing definitions from various countries and regions, our manuscript captures the broader landscape of RD and OD classifications and their implications for healthcare policy, drug access, and health equity.

Moreover, unlike previous works, which primarily highlight the need for harmonization within limited regions, our manuscript advocates for a balanced framework that combines global standardization with region-specific flexibility, a perspective that has not been thoroughly explored. This approach not only enhances our understanding of RD and OD definitions on a global scale but also provides actionable insights for policymakers seeking to balance international consistency with local healthcare needs. Thus, our manuscript adds a unique and valuable perspective, positioning it as a foundational study for advancing both global and regional strategies in rare disease policy and orphan drug accessibility.

Element	our Manuscript (Global Insight into Rare Disease and Orphan Drug Definitions)	Haendel et al. (2020) - “How many rare diseases are there?”[4]	Giannuzzi et al. (2017) - “Orphan medicinal products in Europe and United States”[5]	ISPOR (2015) - “Rare Disease Terminology and Definitions”[6]
Aim	To explore global definitions and classifications of RDs and ODs, and their implications for healthcare policy and access.	To estimate the number of rare diseases and highlight terminological inconsistencies in databases.	To evaluate the status of orphan drug designations and regulatory discrepancies in the EU and US.	To provide an overview of global RD definitions, focusing on terminological and prevalence diversity.
Scope	Broad, with international focus on definitions across multiple regions and their impact on health equity and drug access.	Focuses on quantifying rare diseases and database discrepancies using ontology across databases.	Limited to the EU and US, focusing on regulatory practices and drug accessibility.	Global focus on RD definitions, prevalence thresholds, and jurisdictional diversity.
Methodology	Systematic review using databases like Medline, EMBASE, and Scopus.	Computational and ontological analysis of databases like Orphanet, OMIM, and GARD.	Empirical data analysis from orphan drug registries, particularly from FDA and EMA databases.	Systematic search across jurisdictions, with descriptive statistics on definitions and thresholds.
Findings	Found 200+ unique definitions, significant regional variability, and suggested balance of global standards with regional criteria.	Identified ~10,000 rare diseases, noting disparities across databases; advocates terminological harmonization.	Discrepancies in drug designations between EU and US; recommends collaborative regulatory framework.	Wide variation in prevalence thresholds; suggests standardizing objective criteria.
Rigor of Study	High, adhering to PRISMA-P guidelines, with comprehensive review and protocol registration in PROSPERO.	Moderate, with structured database reliance; computationally robust but lacks systematic review.	Moderate, with comprehensive data from registries but limited geographic coverage.	High, with extensive global coverage and robust statistical analysis but limited content analysis depth.
Other Key Information	Advocates for universal and adaptable definitions to support equitable access and policy.	Calls for global terminological consistency to improve data integration and healthcare outcomes.	Highlights need for EU-US collaboration to improve patient access across regions.	Emphasizes the need for standardized prevalence thresholds for policy alignment and drug access.

Comment 2: ***[There are well known differences in definitions; for instance, the USA, a RD is defined as one that affects fewer than 200,000 Americans, while in Europe and many other countries, a RD is defined as one that affects fewer than 1 in 2000 individuals. It would be interesting to hear about what the actual consequences of these differences have been in the past decades.]***

Response: Thank you, for your insightful comment. Our study addresses these definitional disparities by exploring the consequences of these variations on healthcare access, policymaking, and OD development globally.

These differing definitions influence several key areas: first, they impact patient eligibility for support programs and treatments, with varying thresholds potentially excluding patients in regions with more restrictive definitions. Second, these disparities affect pharmaceutical investment in OD development, as different criteria determine the market size and economic incentives in each region. Finally, regulatory inconsistencies hinder the harmonization of RD and OD policies, which complicates cross-border clinical trials, research collaboration, and access to treatment for rare disease patients.

To address your suggestion, we will incorporate a more detailed analysis of the historical consequences of these definitional differences in our discussion section **highlighted in red** for easy reference, and it present in between **lines 342-348.**

Changes:

Additionally, we observe that historical differences in definitions have had tangible consequences on healthcare outcomes and drug development priorities over recent decades. For instance, the variation in prevalence thresholds between the USA (fewer than 200,000 individuals) and the EU (fewer than 1 in 2,000) has influenced patient eligibility for support and access to treatments, with different thresholds potentially limiting access in regions with more restrictive definitions. These discrepancies have also shaped pharmaceutical investment strategies, as varying definitions impact the perceived market size and economic feasibility of developing treatments for rare diseases in different regions.

Comment 3: ***[The authors write that "subjective standards may result in inconsistent results and implementation challenges. For comprehensive definitions of RDs, ODs, and their subtypes, it is better to combine qualitative and quantitative criteria to be reviewed and updated periodically." While this seems plausible, so many factors go into the quality of health care and research on RDs, that it is not obvious that the definition of what an RD is actually is a major factor. For instance, the health care systems in USA and Europe are vastly different, which has major consequences for individuals with RDs. On the other hand, I do not know of cases where a specific disease is called "rare" in the US but not in Europe (or vice versa), and where this has led to specific problems for individuals affected by that disease – it would add a lot of interest to the paper if the authors could summarize evidence about this and provide literature references.]***

Response: Thank you for your valuable feedback. You raise an important point regarding the broader factors that influence healthcare quality , accessibility, and research for RDs and ODs. We have made the requested changes in the manuscript by adding the refernces and **highlighted them in red** for easy reference, and it present in **lines between 376 – 392.**

Changes:

This SLR emphasizes the importance of developing a local definition for each country, regardless of the criteria applied. Subjective qualifiers can occasionally provide additional context or complexity to the description of RDs, ODs, and their subtypes. However, relying too heavily on subjective standards may lead to inconsistent results and implementation challenges. For comprehensive definitions of RDs, ODs, and their subtypes, it is better to combine qualitative and quantitative criteria, which should be reviewed and updated periodically.

Additionally, differences in disease classification across regions can lead to significant disparities in patient care, research funding, and access to treatments. For instance, cystic fibrosis [7] is classified as rare in Europe and North America, where it benefits from orphan drug designations, incentivizing pharmaceutical companies to develop treatments. However, in regions where it is less common, the lack of this classification can limit research initiatives and access to specialized care [8]. Similarly, sickle cell anemia is

considered rare in the US [9] and UK [9] but is more common in parts of Africa [10], the Middle East [10], eastern and southwestern regions of Saudi Arabia [9], where healthcare systems are better equipped to handle it. In contrast, in countries where sickle cell is classified as rare, patients may face limited treatment options and fewer specialists [11]. These examples highlight how the classification of a disease as rare in one country and common in another can lead to inconsistencies in care, treatment availability, and research focus, underscoring the importance of harmonizing definitions across regions.

Comment 4: ***[The author chose to exclude non-genetic RDs (cancer, infections, poisoning), but there is no obvious reason for doing so, since these individuals will experience many of the same problems. The authors should make it clear that they have investigated genetic RDs or should consider describing the differences between genetic and non-genetic RDs.]***

Response: In response to your comment regarding the exclusion of rare cancers from the analysis, we recognise that rare cancers are a critical aspect of RDs. However, for the purposes of this systematic review, we chose to exclude rare cancers to maintain a focused scope and ensure that the analysis remains manageable and relevant to the broader definitions of RDs and ODs. Rare cancers often have distinct clinical, regulatory, and research considerations compared to non-cancerous RDs. For instance, they typically fall under oncology-specific frameworks that include well-established criteria for diagnosis, treatment pathways, and regulatory incentives like OD designation. Adding rare cancers would have made things more complicated and might have taken away from the more important issues surrounding the definition and regulation of RDs that aren't cancer and ODs. Furthermore, previous literature and regulatory frameworks often treat rare cancers separately, and thus, their exclusion allows us to provide a more cohesive analysis of RDs and ODs that are not cancer-specific. Therefore, we made a deliberate methodological choice to exclude rare cancers in order to produce clear and actionable findings within the intended scope of this review. In addtion, in the SLR published protocol I elaborated on the rationale for focusing on genetically RDs I hope this response clarifies the matter further. I added the protocol reference in **line 116** and **highlighted red.**

Changes: Added the protocol reference [3]

Rebuttal References:

1. Aronson J. Rare diseases, orphan drugs, and orphan diseases. *BMJ* 2006;333:127-28. doi: 10.1136/bmj.333.7559.127
2. Fehr A, Prütz F. Rare diseases: a challenge for medicine and public health. *Journal of health monitoring* 2023;8:3-6. doi: 10.25646/11826
3. Abozaid GM, Kerr K, McKnight A, et al. Criteria to define rare diseases and orphan drugs: a systematic review protocol. *BMJ Open* 2022;12(7):e062126. doi: 10.1136/bmjopen-2022-062126 [published Online First: 20220729]
4. Haendel M, Vasilevsky N, Unni D, et al. How many rare diseases are there? *Nat Rev Drug Discov* 2020;19(2):77-78. doi: 10.1038/d41573-019-00180-y
5. Giannuzzi V, Conte R, Landi A, et al. Orphan medicinal products in Europe and United States to cover needs of patients with rare diseases: an increased common effort is to be foreseen. *Orphanet J Rare Dis* 2017;12(1):64. doi: 10.1186/s13023-017-0617-1 [published Online First: 20170403]
6. Richter T, Nestler-Parr S, Babela R, et al. Rare Disease Terminology and Definitions-A Systematic Global Review: Report of the ISPOR Rare Disease Special Interest Group. *Value in health : the journal of the International Society for Pharmacoeconomics and Outcomes Research* 2015;18:906-14. doi: 10.1016/j.jval.2015.05.008
7. Mehta G, Macek M, Mehta A. Cystic fibrosis across Europe: EuroCareCF analysis of demographic data from 35 countries. *Journal of Cystic Fibrosis* 2010;9:S5-S21. doi: https://doi.org/10.1016/j.jcf.2010.08.002
8. Bell SC, Mall MA, Gutierrez H, et al. The future of cystic fibrosis care: a global perspective. *The Lancet Respiratory Medicine* 2020;8(1):65-124. doi: 10.1016/S2213-2600(19)30337-6
9. Bin Zuair A, Aldossari S, Alhumaidi R, et al. The Burden of Sickle Cell Disease in Saudi Arabia: A Single-Institution Large Retrospective Study. *Int J Gen Med* 2023;16:161-71. doi: 10.2147/ijgm.S393233 [published Online First: 20230113]
10. Moeti MR, Brango P, Nabyonga-Orem J, et al. Ending the burden of sickle cell disease in Africa. *The Lancet Haematology* 2023;10(8):e567-e69. doi: 10.1016/S2352-3026(23)00120-5

11. Bell V, Varzakas T, Psaltopoulou T, et al. Sickle Cell Disease Update: New Treatments and Challenging Nutritional Interventions. *Nutrients* 2024;16(2) doi: 10.3390/nu16020258 [published Online First: 20240115]

VERSION 2 - REVIEW

Reviewer **1**
Name **Giannuzzi, Viviana**
Affiliation **Fondazione per la Ricerca Farmacologica Gianni Benzi ONLUS**
Date **09-Dec-2024**
COI

Dear authors,

thanks for addressing reviewers' comments and for significantly improving the manuscript.

To me, an analysis of regulatory definitions only based on literature still remains a limitation. I understand that the authors set their work on the literature, and this is consistent with the title, aim and methodology; however, as in the regulatory field, the official definitions represent the applicable ones, I consider a limitation to disclose.

The methodological differences between the present analysis and“The protocol for this SLR" should be mentioned in the methods, as well as the reasons for excluding rare cancers.

Reviewer **3**
Name **Robinson, Peter**
Affiliation **The Jackson Laboratory for Genomic Medicine**
Date **27-Nov-2024**
COI

Thank you for the revision, my concerns have been adequately addressed.

VERSION 2 - AUTHOR RESPONSE

Reviewer: 1

Dr. Viviana Giannuzzi, Fondazione per la Ricerca Farmacologica Gianni Benzi ONLUS

Comment 1: To me, an analysis of regulatory definitions only based on literature still remains a limitation. I understand that the authors set their work on the literature, and this is consistent with the title, aim and methodology; however, as in the regulatory field, the official definitions represent the applicable ones, I consider a limitation to disclose.

Response: In light of this valuable feedback, we have revised the "Strengths and Limitations" section of the manuscript to explicitly acknowledge this limitation. We have added the following point to the section between **lines 43-45**, **highlighted in red**.

Changes:

- A limitation of this study is that it relies only on literature-based definitions, which may not fully capture the regulatory definitions officially adopted by agencies, despite these being the ones directly applicable in in real-world situations.

Comment 2: The methodological differences between the present analysis and "The protocol for this SLR" should be mentioned in the methods.

Response: We have incorporated the requested modifications into the manuscript, highlighting the differences between the current analysis and the published protocol **in red**. We have included it in **lines between 141–146** for simple reference.

Changes:

The initial search was conducted in 2021. To ensure the review included the most recent and pertinent studies, updated searches were performed on 31st December 2022 and 31st December 2023. These updates represent a methodological refinement to the original protocol and were undertaken to capture contemporary studies

published after the initial search period. This approach reflects our commitment to ensuring comprehensive coverage of relevant literature and providing the most up-to-date evidence in the analysis.

Comment 3: The methodological differences between the present analysis and "The protocol for this SLR" should be mentioned in the methods, as well as the reasons for excluding rare cancers.

Response: We have incorporated the requested modifications into the manuscript, highlighting the resonse for excluding rare cancer **in red**. We have included it in **lines between 132–139** for easy reference.

Changes:

Rare cancers were excluded from this review to maintain a focused scope and ensure that the analysis remained manageable and relevant to the broader definitions of RDs and ODs. Rare cancers often follow distinct clinical, regulatory, and research frameworks compared to non-cancerous RDs. These include oncology-specific diagnostic criteria, treatment pathways, and regulatory incentives such as OD designation. Including rare cancers would have introduced complexity, potentially detracting from the broader analysis of non-cancerous RDs and ODs. Additionally, rare cancers are frequently treated as a separate category in both regulatory contexts and the literature. Their exclusion aligns with the rationale detailed in the published protocol [1].

Rebuttal References:

1. Abozaid GM, Kerr K, McKnight A, et al. Criteria to define rare diseases and orphan drugs: a systematic review protocol. *BMJ Open* 2022;12(7):e062126. doi: 10.1136/bmjopen-2022-062126 [published Online First: 20220729]
